# Scorched mussels (*Brachidontes* spp., Bivalvia: Mytilidae) from the tropical and warm‐temperate southwestern Atlantic: the role of the Amazon River in their speciation

**DOI:** 10.1002/ece3.2016

**Published:** 2016-02-18

**Authors:** Berenice Trovant, Néstor G. Basso, José María Orensanz, Enrique P. Lessa, Fernando Dincao, Daniel E. Ruzzante

**Affiliations:** ^1^ Instituto de Diversidad y Evolución (IDEAus‐CONICET)Boulevard Brown 2915U9120ACFPuerto MadrynChubutArgentina; ^2^ Departamento de Ecología y EvoluciónFacultad de CienciasUniversidad de la RepúblicaIguá 4225C.P. 11400MontevideoUruguay; ^3^ Universidade Federal do Rio Grande – FURGAv. Itália km 8 Bairro Carreiros96203‐900Rio GrandeBrazil; ^4^ Department of BiologyDalhousie University1355 Oxford St.HalifaxNova ScotiaB3H 4R2Canada

**Keywords:** Amazon River, mussels, southwestern Atlantic ocean, speciation

## Abstract

Antitropicality is a distribution pattern where closely related taxa are separated by an intertropical latitudinal gap. Two potential examples include *Brachidontes darwinianus* (south eastern Brazil to Uruguay), considered by some authors as a synonym of *B. exustus* (Gulf of Mexico and the Caribbean), and *B. solisianus*, distributed along the Brazilian coast with dubious records north of the intertropical zone. Using two nuclear (18S and 28S rDNA) and one mitochondrial gene (mtDNA COI), we aimed to elucidate the phylogeographic and phylogenetic relationships among the scorched mussels present in the warm‐temperate region of the southwest Atlantic. We evaluated a divergence process mediated by the tropical zone over alternative phylogeographic hypotheses. *Brachidontes solisianus* was closely related to *B. exustus I,* a species with which it exhibits an antitropical distribution. Their divergence time was approximately 2.6 Ma, consistent with the intensification of Amazon River flow. *Brachidontes darwinianus,* an estuarine species is shown here not to be related to this *B. exustus* complex. We suspect ancestral forms may have dispersed from the Caribbean to the Atlantic coast via the Trans‐Amazonian seaway (Miocene). The third species*, B rodriguezii* is presumed to have a long history in the region with related fossil forms going back to the Miocene. Although scorched mussels are very similar in appearance, their evolutionary histories are very different, involving major historical contingencies as the formation of the Amazon River, the Panama Isthmus, and the last marine transgression.

## Introduction

A major goal in biogeography has long been to understand the relative roles of historical contingencies vs. contemporary ecological processes in determining the presence/absence of a species in a given geographic region or, more generally, to understand the processes responsible for the geographic distribution of species. The biogeography of South American marine biota is known to have been influenced by major historical contingencies, starting with the separation from Africa (beginning in the Cretaceous) and the opening of the Drake Passage during the middle Eocene (Sanmartin and Ronquist 2004; Scher and Martin 2006), following with the late Eocene opening of the Tasman gateway (Nelson and Cooke 2001) and the subsequent (Oligocene) establishment of a full circum‐Antarctic circulation (west wind drift, WWD). Superimposed on these global processes, there were also a number of processes that affected biodiversity on a regional scale. These include several Atlantic marine transgressions (Malumián and Náñez 2011), the start of the Amazon River outflow toward the Atlantic Ocean beginning during the middle to late Miocene (Figueiredo et al. 2009), the formation of the Panama Isthmus in the Pliocene (Lessios 2008), as well as the glaciations of the Plio‐Pleistocene (Fraser et al. 2012).

All these processes left a significant imprint on the biogeography of the marine biota of South America. The break‐up of Gondwanaland helps explain the transoceanic disjunct distributions of many Southern Hemisphere taxa by vicariant isolation of ancestral lineages (Croizat et al. 1974). On the other hand, the presence of the same species or of closely related taxa throughout a number of subantarctic islands and including the southern tips of continental masses of Australia and New Zealand, South America, and South Africa can best be explained by dispersion along the WWD (Waters 2007; Fraser et al. 2012). For instance, both processes, dispersion prior to vicariance (Heads 2005), have been implicated in the distribution of galaxiid fishes throughout the Southern Hemisphere (Burridge et al. 2012).

Several marine transgressions from the Atlantic have flooded South America to varying degrees and at various times beginning in the Maastrichtian–Danian (66–61 Ma). The last one, recorded for the Middle Miocene (Martínez and del Río 2005; Malumián and Náñez 2011), involves a controversial internal marine connection between the Caribbean Sea and the southern Atlantic Ocean (Rasanen et al. 1995; Webb 1995), inference that is supported by biological and paleontological evidence (Pérez et al. 2011; Stampar et al. 2012; but see Wesselingh and Salo 2006; Cooke et al. 2012). Lastly, the glacial cycles of the Plio‐Pleistocene are also known to have affected the biogeography of the marine biota of South America. The glaciations changed the continental margins of the region (Rostami et al. 2000; Hulton et al. 2002) and hence, the dispersal ability of subtidal and intertidal marine biota with the consequent impact on their distributions and genetic structures (Fraser et al. 2012).

**Figure 1:**
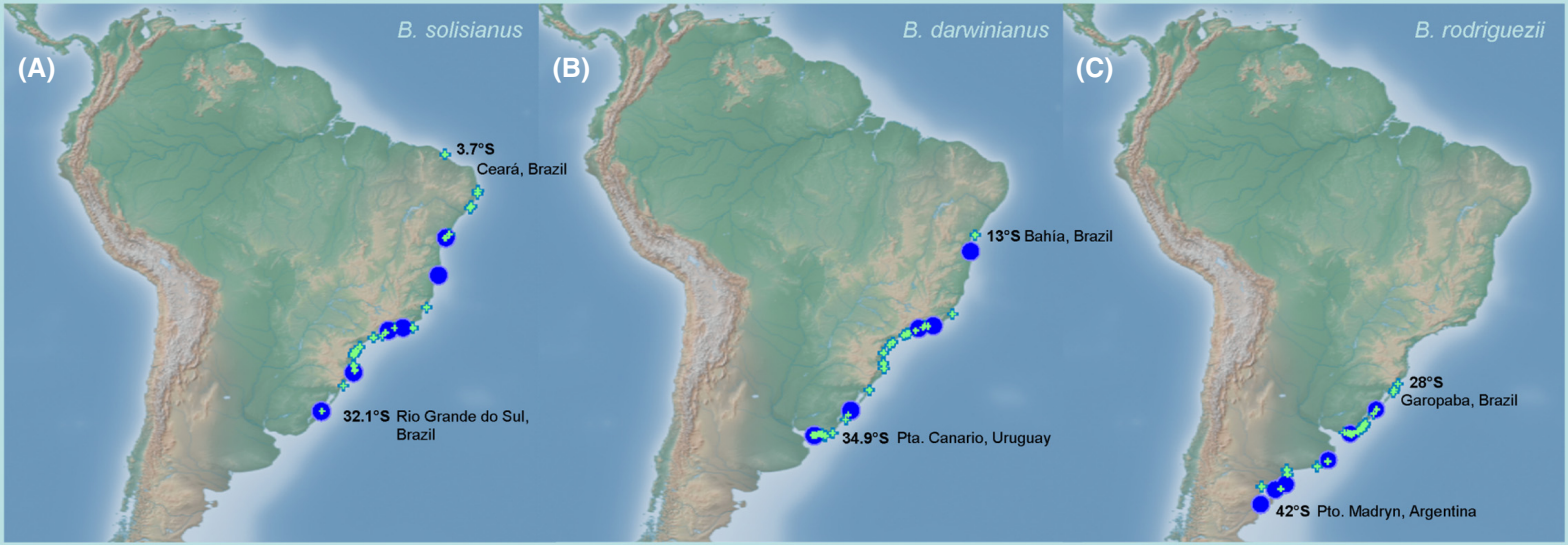
Localities of the genetically analyzed (blue circle) and museum (light green cross) specimens of (A) *Brachidontes solisianus*, (B) *B. darwinianus,* and (C) *B. rodriguezii*, distributed throughout Brazil, Uruguay, and Argentina. See Table 1 for details.

The emergence of the Central American Isthmus affected current flow, salinity, temperature, and primary productivity of the Pacific and the Atlantic Oceans and launched marine organisms of the two oceans into independent evolutionary trajectories (Lessios 2008). For some species, these trajectories ended in extinction while for others they led to the formation of geminate species (Jordan 1908) on both sides of the Isthmus. While these pairs are generally well documented (Lessios 2008), members of potential northwestern and southwestern Atlantic (NW–SW) species pairs are likely underestimated. For example, Vermeij (1991; his Fig. 1), in his global biotic exchange synthesis indicates no trans‐tropical marine exchange in this region. The apparent reason is that compilations of geographic distributions along the shores of the western Atlantic often ignore the intertropical hiatus. Members of such closely related northwestern and southwestern Atlantic pairs are often classified as subspecies or even placed under the same specific name despite being separated by an intertropical latitudinal gap (e.g., Joyeux 2001). This pattern of distribution was named antitropicality (Hubss 1952) and can be divided into three general categories: (1) Strictly bipolar distribution, where related organisms are distributed in cold‐temperate and cold regions of both hemispheres (Ekman 1953). This pattern is well represented among cnidarians (Stepanjants et al. 2006), marine bacteria (Zeng et al. 2010), bryozoans (Kuklinski and Barnes 2010), and protozoa (Darling et al. 2000; Di Giuseppe et al. 2013). (2) Bitemperate distribution (Hubss 1952), where related organisms are distributed in the warm‐temperate regions of both hemispheres. This pattern is well represented among bivalves (Jozefowicz and O′ Foighil 1998; Hilbish et al. 2000; Shilts et al. 2007), fishes (Grant and Leslie 2001), bryozoans (Schwaninger 2008), and starfishes (Nakamura et al. 2011). (3) Distribution interrupted only in the equatorial region, with closely related species present in the intertropical belt. Randall (1982) introduced the term “antiequatorial” to describe this pattern, which is well represented among western Atlantic species or species pairs distributed northwest and southeast of the combined plume of the Orinoco and Amazon rivers, including some reef fishes (Joyeux 2001; Luiz et al. 2013), lobsters (Rodríguez Rey 2010), and crabs (Tourinho et al. 2012).

The suite of historical events described above defines the scenarios in which the phylogenetic and phylogeographic relations of South American marine taxa can be considered. The scorched mussels of the genus *Brachidontes* s.s. (Brachidontinae, including *Mytilaster*, Huber 2010) are a good model for studying this subject. These mussels are ubiquitous in the intertidal zone of rocky shores along both coasts of South America (Tanaka and Magalhães 2002; Bertness et al. 2006; Adami et al. 2013). Four species of brachidontes occur along the Atlantic coast of South America. Three of them (*Brachidontes solisianus*,* B. darwinianus,* and *B. rodriguezii)* are found in warm‐temperate waters, and the fourth (*Perumytilus purpuratus*) is found exclusively in the southern, cold‐temperate waters of southern South America. *Brachidontes solisianus* is a marine species distributed from Rio de Janeiro (22°S) south to Santa Catarina (27°S; Brazil). *Brachidontes darwinianus,* an estuarine species, is distributed from southern Brazil, where it forms mixed beds with *B. solisianus* in regions of low salinity, to the northern coast of the La Plata River estuary (Uruguay). The third species, *B. rodriguezii*, a marine species, extends from Rio Grande do Sul (Brazil) (~32°S; Scarabino et al. 2006; Trovant et al. 2013) south to the North Patagonian gulfs (~43°S Nuevo, San José, San Matías) where it coexists with the fourth species *Perumytilus purpuratus* (Scarabino 1977). *Perumytilus purpuratus* in turn is distributed from 41°S on the Atlantic, south through southern South America and continues on the Pacific side where it is found both in the cold‐temperate and in the warm‐temperate waters of the Magellanic and Chile‐Perú biogeographic provinces, respectively (Briggs and Bowen 2012; Fig. 1) up to Lat. 3°S.

The historical processes likely responsible for the present distribution of these four scorched mussel species vary across species. *Perumytilus purpuratus,* the southernmost species is closely related to *Austromytilus rostratus*, from Australia. The presence of *Perumytilus purpuratus* in South America can thus best be explained by dispersal in an early WWD scenario, following the breakdown of shelf connections between southern Australia, Antarctica, and southern South America during the Eocene (Trovant et al. 2015). The processes that have influenced the distribution for the other three brachidontes remain largely unstudied and require a proper understanding of their phylogenetic relationships. Antitropicality has for instance been signaled as a potential pattern for at least two of the species (*Brachidontes darwinianus* and *B. solisianus*). The phenotype of *Brachidontes darwinianus* (Uruguay and Brazil) is very similar to that of the *B. exustus* species complex (Linnaeus) in the Gulf of Mexico, to the point that some authors consider them synonymous (e.g., Rios 1994). This complex, although, has recently been shown to comprise five cryptic species distributed in the Caribbean and Gulf of Mexico, plus two geminated species in the Pacific (Lee and Ó Foighil 2005). In the absence of genetic studies on *B. darwinianus*, its relationship with the *B. exustus* species complex has thus far remained unresolved. *Brachidontes solisianus,* originally described by d'Orbigny (1842, 1846) based on materials collected in Uruguay (Maldonado) and Brazil (Rio de Janeiro), exhibits numerous records along the Brazilian coast north of Santa Catarina (28°S). This species has also been cited for localities north of the intertropical zone (Rios 1994), although these records require more scrutiny. If confirmed, this pattern would be another potential case of antitropicality. It has also been argued that the third species, *B. rodriguezii*, may actually be related to fossil forms from the Miocene present in the same geographic region where the species is found today (Trovant et al. 2013).

The aim of the present study was to document patterns of antitropical distribution in the western Atlantic region. This general pattern, largely ignored due to perception bias, is frequently observed in many warm‐temperate species in the southwest Atlantic. We examined whether the distribution of two *Brachidontes* species in the southwest Atlantic, *B. solisianus* and *B. darwinianus,* fits an antitropical pattern. This in turn requires the clarification of the relationships within *Brachidontes* s.s. The hypothesis considered, implicit in the taxonomic literature, postulates that *B. darwinianus* and *B. solisianus* are members of antiequatorial pairs (distributed northwest and southeast of the combined plume of the Orinoco and Amazon rivers), with geminates belonging to the *B. exustus* species complex and present in the Gulf of Mexico and the Caribbean. This implies that (1) both *Brachidontes darwinianus* and *B. solisianus* belong to the *B*. “*exustus*” clade; and (2) the approximate time of divergence of both species from the *B. exustus complex* corresponds to the beginning of the deposition of sediment and freshwater flow from the Amazon River to the Atlantic at the end of the uplift of the Andes in the Middle–Late Miocene, around 10 Mya.

## Materials and Methods

### Sample collection

Specimens of *Brachidontes solisianus*,* B. darwinianus,* and *B. rodriguezii* were collected from nine localities distributed along the coast of Brazil and Uruguay from Bahía (~14°S) to Montevideo (~34°S, Fig. 1, Table 1). Tissue samples were stored in 95% ethanol. Morphological traits were measured in sampled specimens and in *Brachidontes* material deposited in the “Museu Oceanografico Eliezer Rios”, Rio Grande, Brazil, and “Museo Nacional de Historia Natural”, Montevideo, Uruguay (Fig. 1, Data S3). To support the assignment of individuals to species, high‐quality images were obtained of the type material of *B. solisianus B. darwinianus*,* B. rodriguezii,* and *B. adamsianus* deposited in the British Museum of Natural History. The phenotypes of the specimens from the Gulf of Mexico, the Caribbean, and the eastern tropical Pacific included in the study of Lee and Ó Foighil (2004, 2005), which had not been adequately described, were properly characterized using material on loan of *B. “adamsianus I*” (*n* = 35), *B*. “*adamsianus II*” (*n* = 3), and *B. “ exustus I*” (*n* = 34), and both valves of each specimen were photographed.

**Table 1:** Sampling sites of *Brachidontes* s.s. species from the coast of Brazil (BR) and Uruguay (UY). In the analysis were added, for comparison, samples from Argentina and Uruguay analyzed in Trovant et al. (2013) (Table 1) indicated by (*)

Species	Locality	Latitude/Longitude
*Brachidontes solisianus*	Itapoa, Bahía (BR)	12°58′S, 38°22′W
	Cumuruxatiba, Bahía (BR)	17°05′S, 39°11′W
	Niteroi, Bahía de Guanabara, Río de Janeiro (BR)	22°52′S, 43°06′W
	Paraty, Río de Janeiro (BR)	23°12′S, 44°43′W
	Praia da Cima, Santa Catarina (BR)	28°00′S, 48°35′W
	Praia do Casino (Navío Altair), Río Grande do Sul (BR)	32°11′S, 52°09′W
*Brachidontes darwinianus*	Bahía de Ilheus, Bahía (BR)	14°47′S, 39°01′W
	Niteroi, Bahía de Guanabara, Río de Janeiro (BR)	22°52′S, 43°06′W
	Paraty, Río de Janeiro (BR)	23°12′S, 44°43′W
	Praia do Casino (Navío Altair), Río Grande do Sul (BR)	32°11′S, 52°09′W
	Punta Canario, Montevideo (URY)*	34°51′S, 56°09′W
*Brachidontes rodriguezii*	Praia do Casino (Navío Altair), Río Grande do Sul (BR)	32°11′S, 52°09′W
	Santa Clara del Mar, Buenos Aires (AR)*	37°50′S, 57°30′W
	Bahía San Blas, Buenos Aires (AR)*	40°32′S, 62°15′W
	Bahía Rosas, Río Negro (AR)*	41°01′S, 64°06′W
	Puerto Madryn, Chubut (AR)*	42°46′S, 65°00′W
*Brachidontes exustus*	Laguna de Chacopata, Sucre (VE)	11°7′N, 64°28′W

DNA sequences of related taxa were downloaded from GenBank and are listed on Table 2, along with the sequences obtained for this study. A nuclear sequence of the 18S rDNA gene available in Genbank identified as *B. dominguensis* Lamarck 1819 was included in the phylogeny taking into account that this species has been considered a junior synonym of *B. exustus* (Rios 2009).

**Table 2:** DNA sequences used in this study and their Genbank access numbers

Species	Locality	COI mtDNA	28S rDNA	18S rDNA	References
*Brachidontes adamsianus I*	Puerto Vallarta, México	AY825158.1‐ AY825170.1	AY825091.1	–	Lee and Ó Foighil (2005)
	Bique Beach, Panamá	AY825181.1, AY825183.1‐AY825187.1	AY825086.1	–	Lee and Ó Foighil (2005)
	Naos, Panamá	AY825173.1‐ AY825180.1	AY825092.1	–	Lee and Ó Foighil (2005)
	Cuastecomate, México	AY825171.1, AY825172.1	–	–	Lee and Ó Foighil (2005)
*Brachidontes adamsianus II*	Isla Jicarón, Panama	–	AY825100.1	–	Lee and Ó Foighil (2005)
*Brachidontes domingensis sensu* Distel (2000)	Coral reef, Dominican Republic	–	–	AF117736.1	Distel (2000)
*Brachidontes darwinianus*	Niteroi, Bahía de Guanabara, Brazil	KT318211‐ KT318214	KT192125‐ KT192126	–	This study
	Bahía de Ilheus, Brazil	KT318202‐ KT318210	KT192127‐ KT192128	KT192099	This study
	Paraty, Brazil	KT318196‐ KT318201, KT318213	KT192107‐ KT192108	KT192095‐ KT192096	This study
	Praia do Casino, Brazil	KT318212‐ KT318215	KT192129‐ KT192130	KT192100‐ KT192101	This study
	Punta Canario, Uruguay	KC844407.1‐KC844414.1	KC844370.1, KC844371.1	KT192097‐ KT192098	Trovant et al. (2013); This study
*Brachidontes erosus*	Taroona	–	KJ453827.1	KJ453810.1	Trovant et al. (2015)
*Brachidontes exustus complex I* Western Caribbean Clade	Bocas del Toro (Caribbean Basin)	AY825127.1 ‐ AY825140.1	AY825084.1	AF229623.1 (USA: Florida, Pinellas County)	Lee and Ó Foighil (2005); Campbell (2000)
	Veracruz (Caribbean Basin)	AY825216.1	AY825104.1		
*Brachidontes exustus complex II* Gulf Clade	Panacea	AY621900.1	AY621999.1		
*B. exustus complex II* Atlantic Clade	Bocas del Toro	AY825140.1	AY825083.1		
*B. exustus complex III* Bahamas Clade	Boca Chica Key, Florida, USA	AY621946.1	AY621992.1	KT318263‐ KT318264 Laguna de Chacopata (Venezuela)	
*B. exustus complex III* Antilles Clade	La Habana, Cuba	AY825154.1	AY825090.1		
*B. granulatus*	Coquimbo, Chile	KJ453888.1; KT318184‐KT318190	KJ453823.1, KT192106	–	Trovant et al. (2015); This study
*B. modiolus*	Florida to Caribbean	AY825218.1	AY622002.1	–	Lee and Ó Foighil (2004)
*B. mutabilis*	Japan: Okinawa, Miyako island	–	AB103124	AB201233.1	Owada (2007)
*B. pharaonis s.l*.	Italy (Mediterranean & Red Sea clade ‐L) Egypt (Safaga/Red Sea)	AY129566.1 (M17)	AJ307536.1.	AJ389643.1	Terranova et al. 2007; Hammer (2001); Steiner and Hammer (2000)
*B. pharaonis s.l*.	Italy (Mediterranean & Red Sea clade ‐M)	AY129565.1 (M11)		–	Terranova et al. (2007)
*B. “variabilis o semistriatus” (pharaonis s.l.)*	Indian Ocean clade	DQ836020.1 (Haplotype II)	AY825099.1 (*B. semistriatus*)	–	Terranova et al. (2007); Lee and Ó Foighil (2005)
*B. “variabilis o semistriatus” (pharaonis s.l.)*	Indian Ocean clade	DQ836019.1 (Haplotype I)		–	
*B. “variabilis” (pharaonis s.l.)*	Pacific Ocean clade	DQ836021.1		–	Terranova et al. (2007)
*B. “variabilis” (pharaonis s.l.)*	Pacific Ocean clade	–	AY825102.1	–	Lee and Ó Foighil (2005)
*B. puniceus*	Cape Verde Islands	HM999785.1		–	Cunha, R.L., Lopes, E. and Castilho, R. (unpublished)
*B. rodriguezii*	Santa Clara del Mar, Argentina	KC844454.1‐KC844459.1	KC844362.1	–	Trovant et al. (2013), This study
	Bahia San Blas, Argentina	KC844460.1‐KC844467.1	KC844363‐KC844367	–	Trovant et al. (2013)
	Bahia Rosas, Argentina	KC844468.1‐KC844476.1	KC844368‐KC844369	–	Trovant et al. (2013)
	Puerto Madryn, Argentina	KC844477.1‐KC844484.1	KC844372‐KC844373	–	Trovant et al. (2013)
	Praia do Casino, Brazil	KT318191‐ KT318195	KT192120‐ KT192123	KT192102‐ KT192104	This study
*B. semilaevis*	Chumical, Panama (Bahamas Clade)	AY825117.1	AY825089.1	–	Lee and Ó Foighil (2005)
*B. solisianus*	Itapoa, Bahía, Brazil	KT318229‐ KT318234	KT192113‐ KT192114	KT192088‐ KT192090	This study
	Cumuruxatiba, Bahía, Brazil	KT318239‐ KT318249	KT192117‐ KT192118	KT192091‐ KT192092	This study
	Niteroi, Bahía de Guanabara, Río de Janeiro, Brazil	KT318235‐ KT318238, KT318261	KT192115‐ KT192116	–	This study
	Paraty, Río de Janeiro, Brazil	KT318216‐ KT318218, KT318262	KT192109‐ KT192110	KT192084‐ KT192085	This study
	Praia da Cima, Santa Catarina, Brazil	KT318219‐ KT318228	KT192111‐ KT192112	KT192086‐ KT192087	This study
	Praia do Casino (Navío Altair), Río Grande do Sul, Brazil	KT318250‐ KT318257	KT192119, KT192124	KT192093‐ KT192094	This study
*Brachidontes sp. 2*	Darwin Hbr, Australia	–	AY825080.1	–	Lee and Ó Foighil (2005)
*Brachidontes sp.1*	Darwin Hbr, Australia	–	AY825081.1	–	Lee and Ó Foighil (2005)
*Brachidontes sp.1 NS‐ morph*	Palau:Ngermeuangel Island, Uet era Ngermeuangel	AB509361.1	–	AB519058.1	Goto et al. (2011)
*Brachidontes sp.2 ON‐morph*	Palau:Ongael Island, Ongael Lake	AB465574.1	–	–	Goto et al. (2011)
*Brachidontes sp.3 MC‐morph*	Palau:Mecherchar Island, Clear Lake	AB465569.1	–	–	Goto et al. (2011)
*Geukensia demissa*	LaHave River estuary, Nova Scotia, Canada (18s). Florida, USA	U56844.1	AY622004.1	L33450.1	Hoeh et al. (1998); Kenchington et al. (1995); Lee and Ó Foighil (2004)
*Geukensia granosissima*	Marco, Florida (COI) ‐ Bradenton, FL, USA	AY621926.1	AY622006.1	–	Lee and Ó Foighil (2005, 2004)
*Ischadium recurvum*	Florida, USA	AY621928.1	AY622008.1	–	Lee and Ó Foighil (2004)
*Mytilisepta virgata*	Shek O, Hong Kong	–	KJ453832.1	KJ453816.1	Trovant et al. (2015)
	Japan:Kanagawa, Manazuru, Shiraiso (COI), Okinawa Prefecture, Japan (28s and 18s)	AB076941.1	KJ453833.1	KJ453817.1	Matsumoto (2003); Trovant et al. (2015)
*Mytilisepta bifurcata*	USA			KJ453814.1‐KJ453815.1	Trovant et al. (2015)
*Perumytilus purpuratus‐South Clade*	Puerto Madryn, Chile	KC844415.1	KC844374.1	–	Trovant et al. (2013)
	Camarones, Chile	KC844419.1	KC844378.1	–	Trovant et al. (2013)
	Pto Deseado, Chile	KC844429.1	KC844386.1	KJ453819.1	Trovant et al. (2013, 2015)
	Ushuaia, Chile	KC844452.1	KC844385.1	–	Trovant et al. (2013)
	Surfer Bay	KC844436.1	KC844387.1	–	Trovant et al. (2013)
	Chiloé, Chile	–	–	KJ453818.1	Trovant et al. (2015)
	Valdivia, Chile	KJ453878.1	KJ598044.1	–	Trovant et al. (2015)
*Perumytilus purpuratus‐North Clade*	San Marcos, Iquique, Chile	KJ453836.1	KJ453825.1	KJ453820.1	Trovant et al. (2015)
	La Chimba, Antofagasta, Chile	KJ453847.1	KJ453826.1	–	Trovant et al. (2015)
	Coquimbo, Chile	KJ453858.1	KJ598048.1	–	Trovant et al. (2015)
	Concepción, Chile	KJ453869.1	KJ598050.1	–	Trovant et al. (2015)
*Austromytilus rostrata*	Taroona, Australia	KJ453834.1	KJ453828.1	KJ453811.1, KJ453812.1, KT192105.1	Trovant et al. (2015)
*Mytilus edulis*	–	NC_006161.1	Z29550.1	L33448.1	Hoffmann et al. 1992; Littlewood 1994; Kenchington et al. 1995;
*Mytilus galloprovincialis*	Japan (28s), Japan:Kanagawa, Ooiso (COI)	AB076943.1	AB105357.1	L33451.1	Matsumoto (2003); Hosoi et al. (2004); Kenchington et al. (1995)
*Atrina pectinata*	Croatia:Rovinj, Northern Adriatic (28s), Japan: Kanagawa, Yokohama Central Market (COI)	AB076914.1	AJ307557.1	EF613241.1	Matsumoto (2003); Hammer (2001); Wang, Z. and Gao, L. (unpublished data)
*Crassostrea gigas*	Japan:Okayama, Ushimado (28s, o 18S) Canada: British Columbia, Nanaimo, shore (COI)	KF644048.1	AB105362.1	AB064942.1	Layton et al. (2014); Hosoi et al. (2004); Itoh, N., Iwashita, M. and Ogawa, K. (unpublished)

### DNA extraction, amplification, and sequencing

DNA was isolated from the posterior adductor muscle using the phenol–chloroform protocol (modified from Sambrook et al. 1989). We used LCO1490/HCO2198 (Folmer et al. 1994), CO1aF/CO1aR (Trovant et al. 2013), and a new set of primers: COI‐UY‐79‐F (5′ ACA AAT CAT AAA GAT ATT GGT ACH YTW TA) and COI‐UY‐iv‐733‐R1 (AAC AAR TGT ATA AAT AAM ACA GGA TC) (Lessa E. and Tomasco I., Universidad de la República, Uruguay) to amplify the cytochrome oxidase subunit I (COI), of 559 bp (aligned length) and D23F/D6R (Park and O′ Foighil 2000) and 22F*/* 1789R (Medlin et al. 1988) to amplify two nuclear genes: the large ribosomal subunit (28S), of 813 bp (aligned length), and the small subunit rDNA (18S), of 1627 bp (aligned length). Additional primers, 18S‐1F 18S‐2F, 18S‐3F; 18S‐4F; 18S‐1R, 18S‐2R, and 18S‐3R (Goto et al. 2011), were used for sequencing the 18S rDNA gene. When possible, we sequenced ten specimens per locality for COI and two specimens per locality for 28S and 18S. In total, we obtained 129 sequences (82 for the COI, 26 for the 28S, and 21 for the 18S). Fewer nuclear than mitochondrial sequences were obtained because of the relatively low variability found in the 28S and 18S nuclear genes. To amplify the genes, we used Tsg polymerase (Bio Basic Inc., Canada). The protocol included an initial denaturing temperature of 95˚ C for 5 min, followed by 30 cycles of 95˚ C for 45 sec, an annealing temperature of 45°C for 1 min for the COI and 52°C for the 28S and 18S, 72°C for 1 min, and a final extension at 72°C for 10 min. After extraction and amplification, the DNA was visualized by UV transillumination in 1% agarose gels stained with fluorescent green dye (BIOTIUM). Extractions and amplifications of DNA samples were performed in the Laboratory of Molecular Biology (CENPAT, Argentina), while the purification of PCR products and sequencing of both strands of DNA were carried out in CENPAT laboratory and Macrogen Inc. (Maryland, USA), using for the COI and the 28S the same primers as in the amplification. DNA sequence data were edited in CodonCode Aligner v 2.0.4 and aligned using default parameters with Clustal W (Thompson et al. 1994).

Some Mytilids have a form of mtDNA inheritance known as “doubly uniparental inheritance” (DUI) (Fisher and Skibinski 1990; Hoeh et al. 1991; Zouros et al. 1992; Geller 1994; Skibinski et al. 1994a,b; Stewart et al. 1995; Quesada et al. 1996). This phenomenon has been found in some brachidontes (*B. exustus* species complex, Lee and Ó Foighil 2004; *P. purpuratus* Vargas et al. 2015), but not in others (Terranova et al. 2007). Species exhibiting DUI are characterized by two distinct mtDNAs: A maternally inherited mitochondrial genome present in eggs and somatic tissues of females and males and a different, paternally inherited mitochondrial genome in the male germ line (Rawson and Hilbish 1995). The paternal mtDNA is preferentially replicated, particularly in the gonad. Following Lee and Ó Foighil (2004), we extracted DNA from the posterior adductor muscle tissue because this muscle is unlikely to be infiltrated by germ line tissue irrespective of the sex of an individual mussel.

### Phylogenetic analyses and divergence time estimation

To assess the degree of saturation of mitochondrial sequences, a test of substitution saturation (Xia and Lemey 2009) was performed in DAMBE v5 (Xia 2013). Subsequently, two phylogenies of *Brachidontes* s.s were constructed, one based on a concatenated 28S and 18S dataset and the other on COI sequences. Nuclear and mitochondrial analyses were performed separately, due to the large difference in the number of available sequences of terminal taxa. The mytilids *Mytilus galloprovincialis* and *M. edulis* (Mytilinae), *Ischadium recurvum* (as Mytilinae en Huber 2010), *Geukensia* spp. (Brachidontinae), and one representative of each of the genera of the AMP clade (*sensu* Trovant et al. 2015; *Austromytilus*,* Mytilisepta* y *Perumytilus*) were selected as outgroups.

Two methods were utilized for phylogenetic reconstruction: maximum likelihood (ML) and Bayesian inference (BI). The Akaike Information Criterion (AIC), implemented in jModelTest v 2.1.5 (Darriba et al. 2012), was applied to find the models of evolution that best fit the data (see Supporting information, Data S1). The selected models were used in ML analyses of nuclear and mitochondrial datasets, conducted with RAxML 7.4.2 (Stamatakis 2006), and implemented in raxmlGUI 1.3 (Silvestro and Michalak 2012) with 1000 replicates. Phylogenies reconstructed with BI were estimated with different substitution (HKY+G+I, Hasegawa et al. 1985; and GTR+G+I, Tavaré 1985) and tree (Yule and birth–death processes) models. The marginal‐likelihood scores of the posterior distributions were compared using Bayes Factors (BFs, Kass and Raftery 1995) with two different methods: harmonic mean estimation (HME, Newton and Raftery 1994) and a posterior simulation‐based analog of the Akaike information criterion through Markov chain Monte Carlo analysis (AICM, Raftery et al. 2007) implemented in Tracer v1.6 (Rambaut et al. 2014) (see Supporting information, Tables S1.1 and S1.2). Bayesian reconstructions were conducted using BEAST v. 1.8.0 (Drummond et al. 2012) with a Markov chain Monte Carlo (MCMC) simulation for 100 million generations for the nuclear phylogenies and mitochondrial dataset, sampling trees every 1000 generations with a burn‐in of 25%. Convergence diagnostics were conducted in Tracer, and reliable ESS values (>200) were ensured. Then, the maximum credibility tree was generated from the combined trees in TreeAnnotator v 1.6.1 (Drummond et al. 2012). Finally, the edition of the trees was carried out in FigTree v 1.4 (Morariu et al. 2008).

The substitution rate and divergence times among *Brachidontes solisianus* and *B. exustus I* (Western Atlantic Clade) were estimated from the COI dataset with the equation *μ *= (1/2 × d)/t, where *μ* is the substitution rate, d is the distance, and t is the genetic divergence time, using the separation time among *B*. “*exustus I* “ and *B*. “*adamsianus I*” *sensu* Lee and Ó Foighil (2005), which was estimated from the formation of the Isthmus of Panama (3.3 ± 0.2 Ma, Lessios 2008).

### Genetic diversity and population structure

The estimates of evolutionary divergence over sequence pairs within and between *B. solisianus*,* B*. “*exustus I*”, and *B*. “*adamsianus I*”, were calculated using “*p‐distance*” (Kimura 1980) in MEGA v5 (Tamura et al. 2011). Most sampling sites are separated by hundreds of kilometers and were thus considered to represent distinct populations. One exception comprised two sampling sites located in Rio de Janeiro (Niteroi 22°S and Paraty 23°S) that were considered a single locality. Standard diversity indices such as the number of polymorphic sites (S), number of haplotypes (k), haplotype diversity (Hd), nucleotide diversity (*π*), and mean number of pairwise differences (П) were estimated for each population using Arlequin 3.5 (Excoffier and Lischer 2010). In addition, pairwise F_ST_ estimates were obtained for mtDNA COI based on 10,000 permutations with Arlequin. The significance of pairwise comparisons between populations was tested applying Holm–Bonferroni sequential correction (Holm 1979). To represent the spatial distribution of haplotypes, we constructed a maximum‐parsimony COI haplotype network using the median joining algorithm (Bandelt et al. 1999) with default parameters using Network v 4.6.13 (Polzin and Daneschmand 2003). Following the construction of the network, a “MP calculation” was performed to reduce the number of links and unnecessary vectors.

### Demographic history

The hypothesis of neutrality, where a constant population size is assumed, was examined with two different approaches. Fu's *F*_*s*_ (Fu 1997) and Tajima's *D* (Tajima 1989) tests were calculated on COI sequences using DnaSP v 5.10 (Librado and Rozas 2009). In addition, mismatch distribution analysis (Rogers and Harpending 1992) was used to visualize the signature of the expansion, and the Harpending's raggedness index (r) (Harpending et al. 1993) was calculated to quantify the smoothness of the observed distribution. In a population that has been stationary for a long time, these distributions from nonrecombinant DNA sequences become ragged and erratic, whereas a population that has been growing generates mismatch distributions that are smooth and unimodal (Harpending 1994). In expanding populations, the raggedness value is low and nonsignificant, while it is usually high and significant in stationary populations.

## Results

### Phylogeny *Brachidontes* s.s. and divergence time estimation

**Figure 2:**
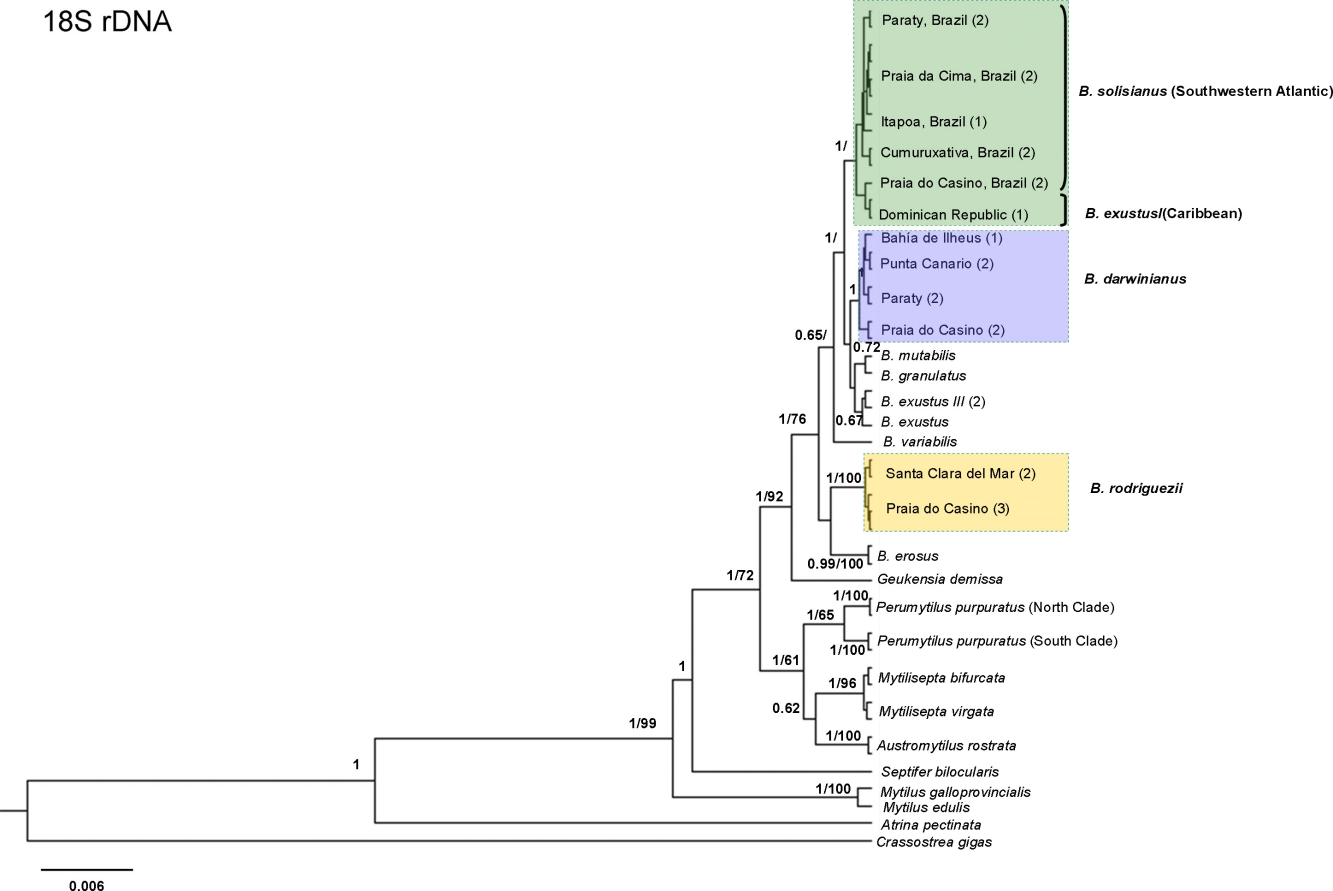
Phylogenetic Bayesian reconstruction of Brachidontinae from the 18S rDNA gene. Numbers on branches represent the values of Bayesian posterior probabilities/bootstraps of maximum likelihood (only >60) as support for nodes. The species sequenced in this study are indicated in bold and numbers in parentheses following the name of the species indicate the number of sequences.

**Figure 3:**
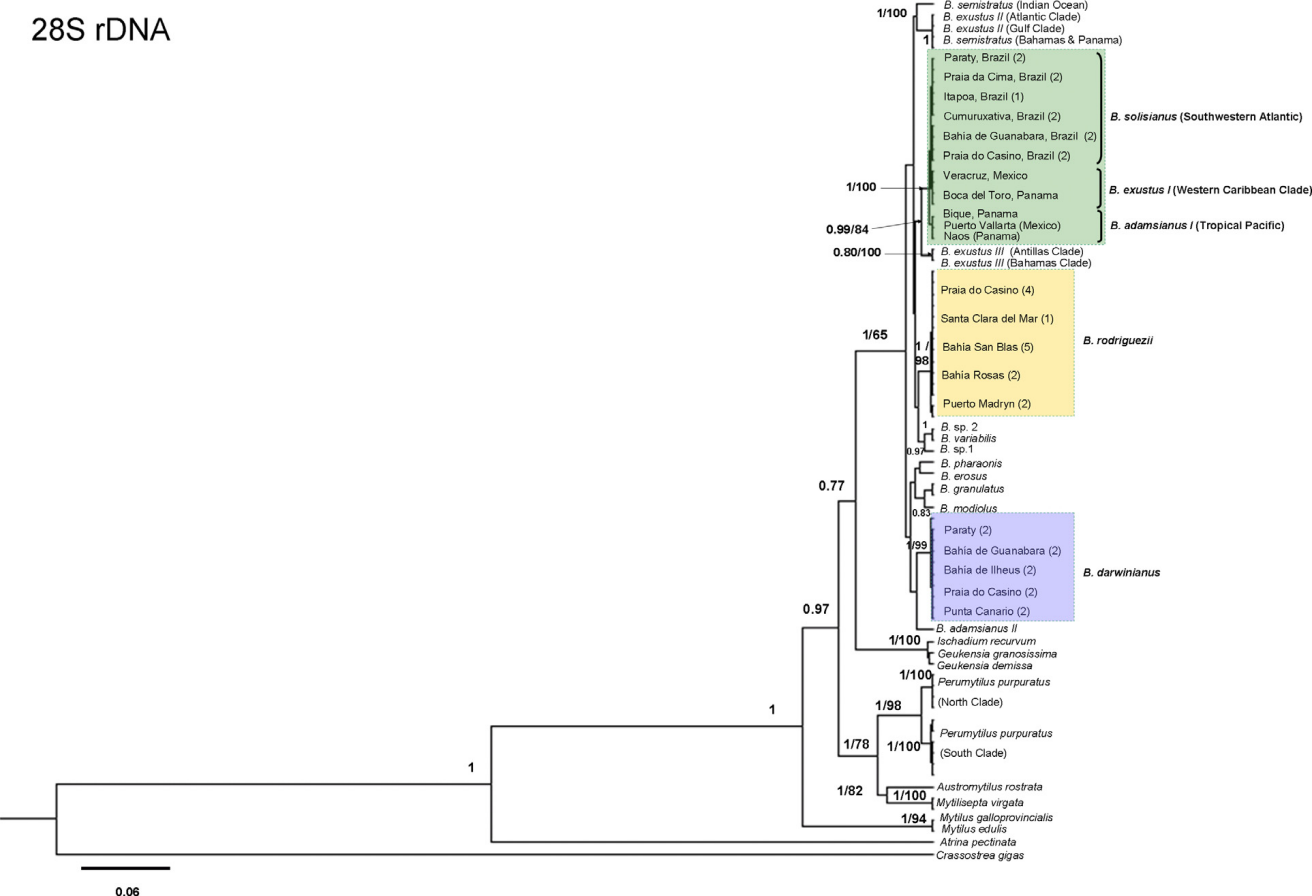
Phylogenetic Bayesian reconstruction of Brachidontinae from the 28S rDNA gene. Numbers on branches represent the values of Bayesian posterior probabilities/bootstraps of maximum likelihood (only >60) as support for nodes. The species sequenced in this study are indicated in bold and numbers in parentheses following the name of the species indicate the number of sequences.

**Figure 4:**
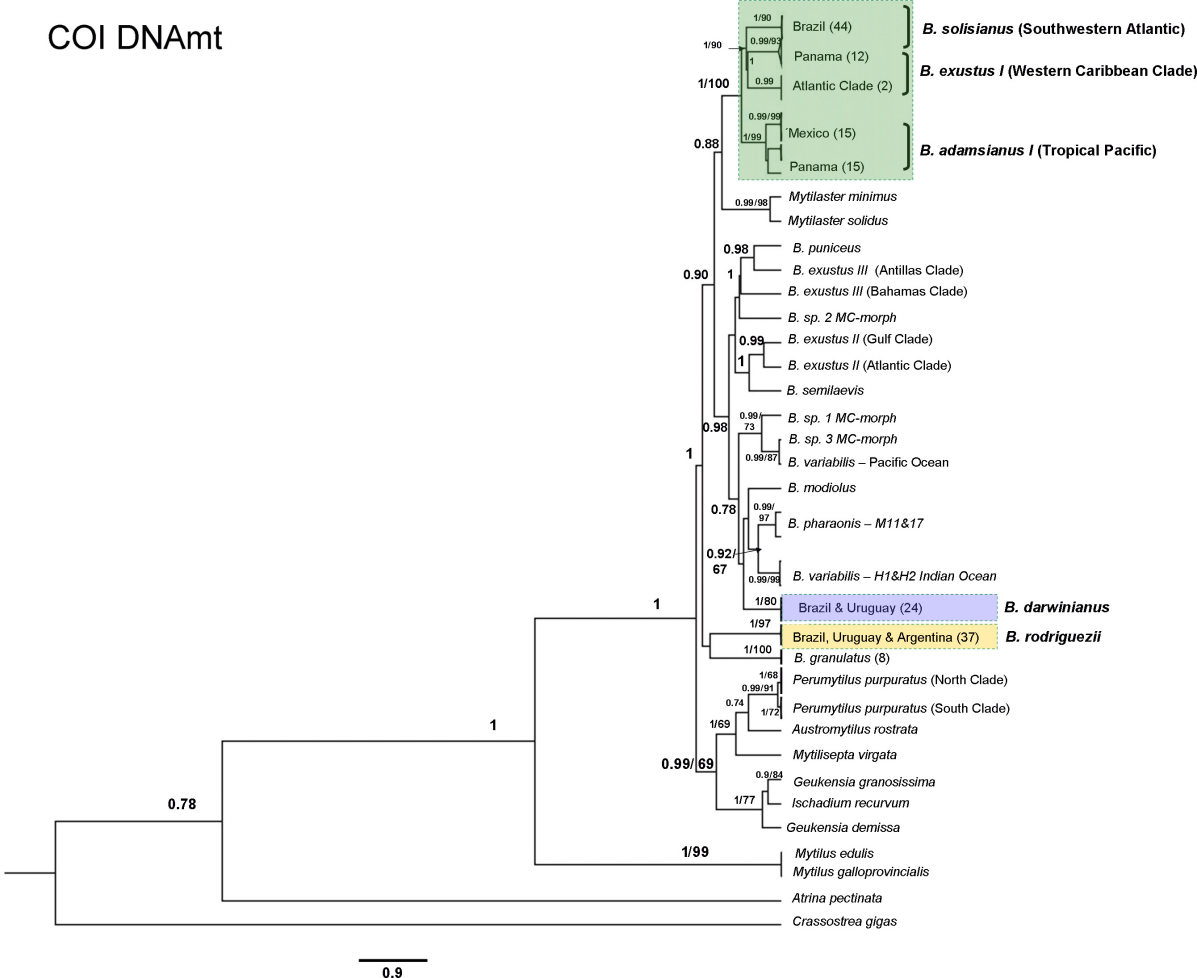
Phylogenetic Bayesian reconstruction of Brachidontinae from mitochondrial COI gene. Numbers on branches represent the values of Bayesian posterior probabilities/bootstraps of maximum likelihood (only >60) as support for nodes. (Numbers in parentheses) Collapsed nodes, the sequences of all sampled locations are included; see details on Tables 1 and 2. The species sequenced in this study are indicated in bold and numbers in parentheses following the name of the species indicate the number of sequences.

The phylogenies were inferred from mitochondrial and nuclear data under the selected substitution model GTR+G+I (for details see Supporting information) and recovered three clades: (1) *Brachidontes* s.l., (2) *Geukensia* + *Ischadium,* and (3) *Austromytilus* + *Mytilisepta* + *Perumytilus* (Figs 2, 3, 4). Within the *Brachidontes* clade, both the nuclear and mitochondrial phylogenies distinguish the three focal species *Brachidontes solisianus, B. darwinianus,* and *B. rodriguezi* with high support. While the relationships among the members of the *Brachidontes* clade, including *B. darwinianus* and *B. rodriguezi,* differ between the mitochondrial and nuclear phylogenies (Figs 2, 3, 4), neither the nuclear nor the mitochondrial genomes place *B. darwinianus* or *B. rodriguezii* in a close relationship with the *B. exustus* species complex. Lastly, whereas both phylogenies group *Brachidontes solisianus* (Brazil) together with *B*. “*exustus I*” (Western Caribbean) and its geminate pair *B*. “*adamsianus I*” (Eastern Pacific), the nuclear phylogeny does not distinguish among these three species. Assuming a divergence time between *B*. “*exustus I*” and *B*. “*adamsianus I*” of 3.3 ± 0.2 Mya following the formation of the Panama Isthmus and a genetic distance 20.2% (see Table 3), we estimated a substitution rate of 0.03 substitutions/site/Myr. Using this substitution rate, we then estimated the divergence time between *B*. “*exustus I*” and *B. solisianus* to be 2.6 ± 0.6 Mya.

**Table 3:** Genetic distances calculated with the “*P*‐distance” method on the COI dataset between and within *Brachidontes solisianus*,* B*. “*exustus I*” and *B*. “*adamsianus I*”, based on mitochondrial sequence data. The estimates of the standard error (SE) were obtained by bootstrap (1000 replicates)

	Mean genetic distance (%)	SE
Between groups		
*B. solisianus‐ B*. “*exustus I*”	16.0	0.015
*B. solisianus‐ B*. “*adamsianus I*”	17.5	0.014
*B*. “*exustus I*”*‐ B*. “*adamsianus I*”	20.2	0.014
Within groups		
*B. solisianus*	0.002	0.001
*B*. “*exustus I*”	0.074	0.007
*B*. “*adamsianus I*”	0.032	0.005

### Genetic diversity, population structure, and demographic history

Observed saturation indices were significantly lower than expected (Isso: 0.22 to 0.23 < Isse: 0.35 to 0.75; *P* < 0.05), suggesting little saturation for mitochondrial sequences; we thus performed all analyses with the full COI dataset. No indels or stop codons were detected in these sequences.

Interspecific genetic distances between *B. solisianus*,* B*. “*exustus I*”, and *B*. “*adamsianus I*” ranged between 16% and 20.2%, while intraspecific genetic distances varied between 0.02% and 0.03% (*N* = 89, Table 3).

Genetic diversity indices were similar between species. Only one species, *B. solisianus*, showed evidence of population expansion and/or positive selection, with significant and negative values of *F*_*s*_ and Tajimaʼs D (Table 4).

**Table 4:** Genetic diversity indexes and neutrality tests by locality and species based on mtDNA sequences (COI) of *Brachidontes* species. N: number of samples; S: number of polymorphic sites; k: number of haplotypes; Hd: haplotype diversity; π: nucleotide diversity; П: average number of nucleotide differences; and SD: standard deviation. (*) Statistically significant differences. Fs is considered significant when *P* < 0.02, while Tajima's D is considered significant when *P* < 0.05

Locality	N	S	k	Hd	π	П	*F*_*s*_	Tajima's D
*B. solisianus*								
Cumuruxativa	11	7	5	0.80	0.002	1.48	−0.66 (*P* = 0.25)	−1.03 (*P* = 0.17)
Niteroi + Paraty	9	8	5	0.83	0.003	2.00	−0.78 (*P* = 0.27)	−1.46 (*P* = 0.09)
Itapoa	6	6	4	0.90	0.003	2.00	−0.56 (*P* = 0.22)	−0.66 (*P* = 0.37)
Praia da Cima	10	9	7	0.86	0.003	1.95	−3.34* (*P* = 0.01)	−1.68* (*P* = 0.03)
Praia do Casino	8	3	2	0.58	0.001	1.11	1.84 (*P* = 0.78)	−1.51 (*P* = 0.05)
Total	44	22	19	0.68	0.002	1.34	−19.41* (*P* = 0.001)	−2.41* (*P* < 0.01)
*B. darwinianus*								
Bahía de Ilheus	9	1	2	0.22	0.0003	0.22	0.67 (*P* = 0.43)	−1.36 (*P* = 0.09)
Niteroi + Paraty	9	3	3	0.41	0.0013	0.66	−0.38 (*P* = 0.17)	−1.51 (*P* = 0.05)
Praia do Casino	2	–	1	–	–	–	–	–
Punta Canario	8	3	4	0.64	0.0015	0.75	−1.83* (*P* = 0.01)	−1.45 (*P* = 0.07)
Total	26	9	9	0.81	0.005	2.21	−0.38 (*P* > 0.10)	−1.08 (*P* = 0.30)
*B. rodriguezii*								
Praia do Casino	6	6	3	0.73	0.009	2.80	1.67 (*P* = 0.80)	0.38 (*P* = 0.65)
Santa Clara del Mar	6	7	3	0.60	0.006	3.33	2.03 (*P* = 0.87)	0.51 (*P* = 0.67)
Bahía San Blas	8	10	5	0.86	0.007	4.04	0.33 (*P* = 0.53)	0.22 (*P* = 0.63)
Bahía Rosas	9	2	3	0.41	0.0008	0.42	−1.08 (*P* = 0.05)	−1.36 (*P* = 0.09)
Puerto Madryn	8	1	2	0.25	0.0004	0.25	−0.18 (*P* = 0.20)	−1.05 (*P* = 0.22)
Total	37	10	7	0.58	0.006	1.92	1.16 (*P* > 0.10)	0.37 (*P* = 0.83)

The paired Φ_ST_ values indicated no significant differentiation among *B. solisianus* populations (Table 5A). In contrast, significant differentiations were found between *B. darwinianus* populations (Table 5B) as well as between some of the *B. rodriguezii* populations (Table 5C).

**Table 5:** Φ_ST_ paired comparisons (below the diagonal) and approximate distance in km (above the diagonal) between populations of (A) *B. solisianus*, (B) *B. darwinianus,* and (C) *B. rodriguezii*. The uncorrected *P*‐values are shown to the left of the bar and the *P*‐values after Bonferroni–Holm correction to the right of the bar. (*): Statistically significant differences (*P*‐value below its corrected value). In *B. darwinianus*,* P*‐values were all equal to 0.0000001 so the Bonferroni–Holm correction could not be calculated

*Brachidontes solisianus*	1	2	3	4	5
1. Cumuruxativa	–	980	1950	2230	2960
2. Niteroi + Paraty	0.008 (0.50/0.010)	–	970	1200	1900
3. Itapoa	0.001 (0.37/0.008)	0.004 (0.35/0.007)	–	290	1000
4. Praia da Cima	0.030 (0.77/0.05)	0.029 (0.73/0.025)	0.028 (0.70/0.017)	–	730
5. Praia do Casino	0.047 (0.14/0.006)	0.006 (0.58/0.013)	0.145 (0.08/0.005)	0.045 (0.11/0.006)	–

*Brachidontes darwinianus*	1	2	3	4
1. Bahía de Ilheus	–	1270	3200	3570
2. Niteroi + Paraty	0.84*	–	1900	2380
3. Praia do Casino	0.93*	0.29	–	400
4. Punta Canario	0.85*	0.68*	0.71*	–

*Brachidontes rodriguezii*	1	2	3	4	5
1. Praia do Casino	–	1100	1700	1800	2100
2. Santa Clara del Mar	0.16 (0.99/0.025)	–	500	600	1100
3. Bahía San Blas	0.30* (0.004/0.006)	0.34* (0.003/0.006)	–	100	400
4. Bahía Rosas	0.61 (0.10/0.02)	0.64* (0.003/0.005)	0.257 (0.01/0.008)		300
5. Puerto Madryn	0.59 (0.014/0.01)	0.62* (0.006/0.007)	0.236 (0.08/0.013)	0.000 (0.99/0.05)	–

**Figure 5:**
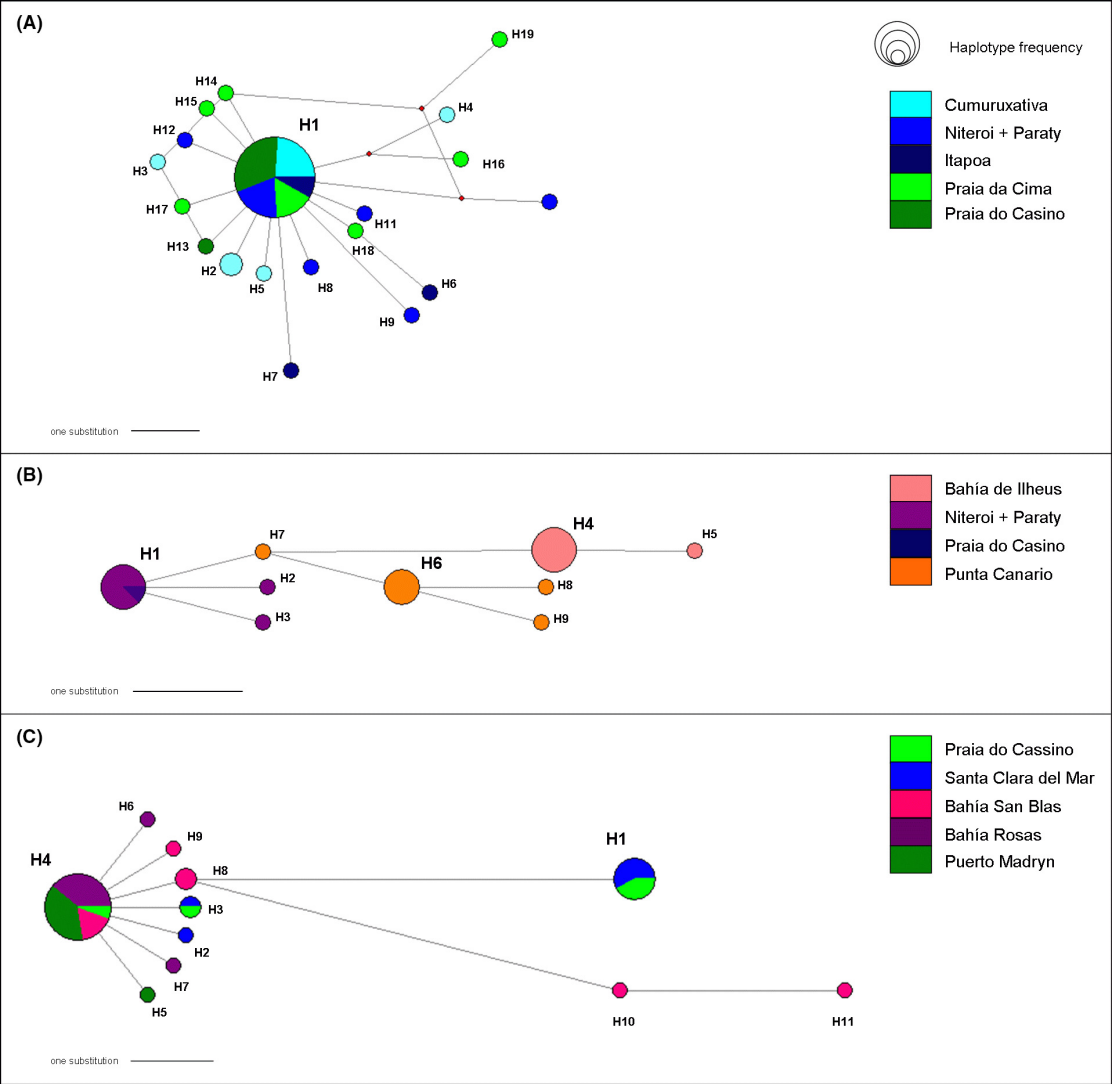
Haplotype networks based on COI of: (A) *B. solisianus*, (B) *B. darwinianus* y, (C) *B. rodriguezii*.

**Figure 6:**
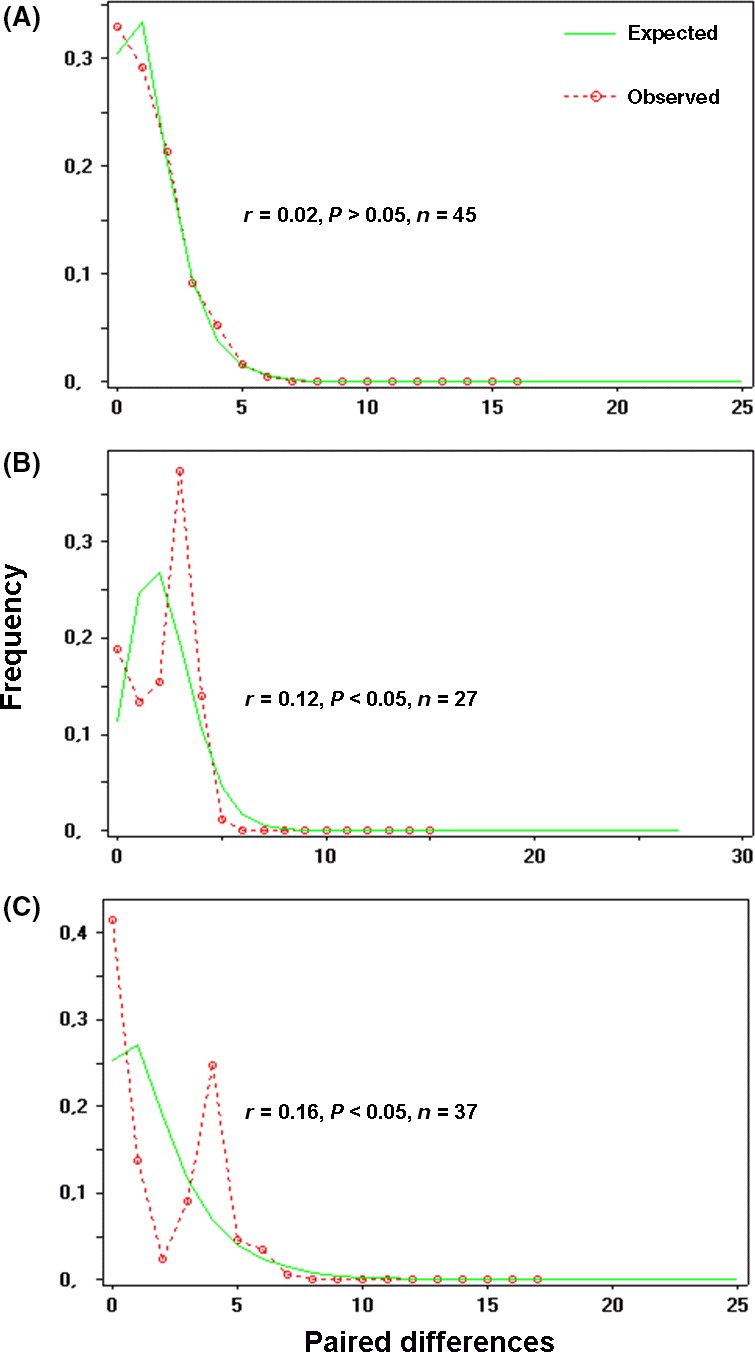
Distribution mismatch of: (A) *B. solisianus*, (B) *B. darwinianus* y, (C) *B. rodriguezii*.

*Brachidontes solisianus* exhibited a star‐like haplotype network, a unimodal distribution, and a low and no significant Harpending index value (Figs 5A and 6A), while *B. darwinianus* and *B. rodriguezii*, both exhibited nonstar‐like or expanded networks, multimodal distributions, and a higher and significant Harpending index value (Figs. 5B,C and 6B,C). In the haplotype network of *B. solisianus*, the haplotype 1 has the highest frequency, a wide geographic distribution and numerous connections with rare haplotypes (Fig. 5A). For *B. darwinianus,* the haplotype 1 and 4 are the most frequent (Fig. 5B), while for *B. rodriguezii* (Fig. 5C) haplotype 4 is the most common and has a broad geographic distribution.

### Review of the museum material

*Brachidontes solisianus* museum samples were distributed from Ceará (Brazil, 3.7°S) to the east dock of Rio Grande do Sul (32.1°S), those of *B. darwinianus* samples were distributed from Bahia (Brazil, 13°S) to Punta del Este (Uruguay, 34.9°S), and those of *B. rodriguezii* were distributed from Garopaba (Brazil, 28.9°S) to Puerto Madryn (Argentina, 42°S) (Fig. 1; Data S2–3).

**Figure 7:**
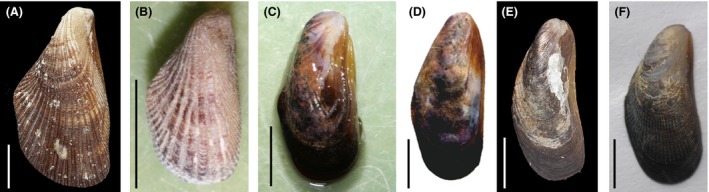
(A) Syntype of *Mytilus adamsianus* Dunker from Panama (ID: 18565317_04, image courtesy of Harry Taylor, Natural History Museum); (B) *B*. “*adamsianus II*” from Jicaron Island, Panama (Lee and Ó Foighil 2005, ID: 9067); (C) *B*. “*adamsianus I*” from Puerto Vallarta, Mexico (Lee and Ó Foighil 2005, ID: 9077); (D) *B*. “*exustus I*” from Veracruz, Mexico (Lee and Ó Foighil 2005); (E) Syntype of *B. solisianus* dˈOrbigny; (F) *B. solisianus* from Cumuruxativa, Brazil.

We re‐examined and characterized the phenotypes of the material identified by Lee and Ó Foighil (2004, 2005) as *B. adamsianus I, B. adamsianus II,* and *B. exustus I*. Based on our photographic comparisons with their type material, we found that specimens they identified as *B. “adamsianus II”* phenotypically match the original description of *B. adamsianus* (Fig. 7A), while the material they identified as *B*. “*adamsianus I* “(Fig. 7C) is phenotypically similar to the type material of *B. solisianus* d'Orbigny (Fig. 7E), the *B. solisianus* specimens we collected (Fig 7F) and *B*. “ *exustus I* “(Fig. 7D).

## Discussion

### Phylogenetic relationships and phylogeographic patterns

Despite the relatively small sample sizes and loci analyzed, we observed a highly supported general pattern. Phylogenetic reconstructions based on nuclear and mitochondrial data suggest that *B. solisianus*,* B. darwinianus,* and *B. rodriguezii* are species belonging to different lineages within the *Brachidontes* s.s. clade. *Brachidontes solisianus* is closely related to *B. exustus I,* a species with which it exhibits an antitropical distribution, and to this species' geminate pair from the Pacific Ocean*, B. adamsianus I. Brachidontes darwinianus,* an estuarine species previously considered on the basis of phenotype to be related to the *B. exustus* complex, is shown here not to be related to this complex. We suspect ancestral forms may have dispersed from the Caribbean to the Atlantic coast via the Trans‐Amazonian seaway (Miocene). The third species*, B rodriguezii*, is presumed to have a long history in the region with related fossil forms going back to the Miocene. Below, we describe the species' distribution patterns and the processes potentially responsible for these distributions in detail.

### *Brachidontes solisianus* and antitropicality

The mitochondrial phylogeny groups *Brachidontes solisianus*, a species distributed along the coast of Brazil, with *B*. “*exustus I*” from the Gulf of Mexico and Caribbean and with *B*. “*adamsianus I*” from the Tropical Eastern Pacific. Thus, while all three species group within the *B. exustus* complex *sensu* Lee and Ó Foighil (2005), their distributions are disjunct. One disjunction, caused by the appearance of Isthmus of Panama, separates *B*. “*exustus I*” from *B*. “*adamsianus I*” (discussed by Lee and Ó Foighil 2005). The second disjunction corresponds to an equatorial gap between *B. “exustus I*” and *B. solisianus*. This disjunction is an example of antitropicality known as “antiequatorial distribution” (Randall 1982), with *B*. “*exustus I*” and *B. solisianus,* respectively, distributed northwest and southeast of the Orinoco and Amazon combined pens.

The antiequatorial patterns observed in the western Atlantic have sometimes been attributed to the flow history of the Amazon. While temperature has been hypothesized to explain the origin of bipolar and bitemperate distributions during the cooling events of the Plio‐Pleistocene (Berg 1933; Grant and Leslie 2001; Burridge 2002), it is unlikely to have been a relevant factor in the distribution of *B. solisianus* and *B*. “*exustus I*” as both species are present in the warm waters of the intertropical region. Instead, salinity and habitat are the likely factors responsible for the disjunction exhibited by these marine rocky shore species. Geological records indicate the deposition of sediment and freshwater flow from the Amazon River to the Atlantic began at the end of the uplift of the Andes in the Middle‐Late Miocene, around 10 Mya (Hoorn 1993, 1996). Currently, the Amazon River discharges a large volume of freshwater into the Atlantic (Curtin 1986a,b) altering salinity and causing sediment discharge up to 500 km from the coast (Rocha 2003). This barrier is considered to be selective as it affects different species to varying degrees. For instance, while some populations or species of reef fish exhibit low genetic differentiation across the barrier (Joyeux 2001), the spiny lobster (*Panulirus argus*) exhibits high genetic differentiation among populations from the Caribbean and Brazil with an estimated time of divergence of 16 Mya (5–23 Mya; Tourinho et al. 2012), a time consistent with the start of the Amazon flow to the Atlantic. The genetic distance found in the lobster is comparable to that found between *Brachidontes solisianus* and *B*. “*exustus I*” in this study.

The divergence time for the pair *B. solisianus*–*B*. “*exustus I*” was estimated at 2.6 Mya (2.45–2.77 Mya), centered at the beginning of the Quaternary. This disjunction would be more recent than the divergence between *B*. “*exustus I*” and *B*. “*adamsianus I*”, attributed to the formation of the Isthmus of Panama, and estimated at 3.3 Mya (3.1–3.5 Mya). The lower genetic distance between *B*. “*exustus I*” and *B. solisianus* than between *B*. “*exustus I*” and *B*. “*adamsianus I*” likely reflects the “permeability” of the Amazon barrier compared to the Isthmus of Panama during the Quaternary rather than the relative age of the geologic event that created the disjunction.

**Figure 8:**
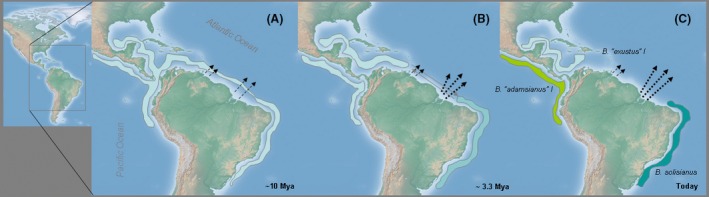
Hypothetical events leading to speciation in *Brachidontes*. (A) Early Late Miocene: the reverse of the flow of the Amazon River to the Atlantic Ocean with the end of the uplift of the Andes generates a permeable barrier to the population of *Brachidontes* sp. with a wide distribution; (B) Pliocene–Pleistocene: After intensifying the flow of the Amazon River (~5 Ma), the differentiation of *Brachidontes solisianus* continued. The formation of the Isthmus of Panamá led to the initiation of the differentiation of *B. adamsianus* I and *B. exustus* I. (C) Actual distribution of the species of *Brachidontes*.

The most plausible hypothesis to explain the evolutionary history of *B. solisianus* and *B. “exustus I*” is vicariance through parapatric speciation (separation with casual contact) (Fig. 8). Prior to the origin of the Amazon (ca. 10 Ma) and the formation of the Isthmus of Panama, the common ancestor of *B. solisianus*,* B*. “*exustus I*”, and *B*. “*adamsianus I*” would have been distributed in the eastern tropical Pacific, Caribbean, and the coast of Brazil. The beginning of the Amazon River flow started to develop a barrier that separated the Brazilian and Caribbean populations, which began to differentiate. The divergence between *B. solisianus* and *B*. “*exustus I*” may have been associated with the intensification of river flow caused by the uplift and erosion of the Andes during the Pliocene (5.3 ± 1.6 Mya, Hoorn 1994), becoming more effective during the Pleistocene. The separation of *B*. “*adamsianus I*” from *B. “exustus I*” was instead, the result of the formation of the Isthmus of Panama, usually estimated at 3.3 Mya.

The age of closure of the isthmus and its biogeographic implications has been subject of recent debate (Stone 2013). The hypothesis with more evidence is its complete closure during the Pliocene (3.1–3.5 Mya, e.g., Jackson and O'Dea 2013) while alternate hypotheses support a much earlier closure (~15 Ma, Montes et al. 2012; Bacon et al. 2015). However, the shallow interoceanic connections appear to have continued during the Pliocene, not affecting the connectivity between populations of species such as small mussels distributed in shallow waters. After the final closure of the isthmus (~3.3 Ma), the populations of the eastern tropical Pacific and the Caribbean were completely isolated, without possibility of gene flow. Thus, while the most probable hypothesis for the speciation of *B*. “*adamsianus I*” (tropical Pacific) is vicariance by allopatry with the formation of the Isthmus of Panama 3.3 Mya, the most probable hypothesis for the speciation of *B. solisianus* is parapatric vicariance, a slow process that may have begun in the Late Miocene with the origin of the Amazon River flow and would have culminated during the Quaternary with the intensification of the current.

### *Brachidontes darwinianus* and the trans‐Amazonian seaway

The hypothesis of an antitropical distribution and a close relationship between *B. darwinianus* and the *B. exustus* complex (based on phenotypic resemblance, Rios 1994) is clearly refuted by our results. The nuclear phylogeny indicates that *B. darwinianus* is not a part of the *B. exustus* species complex. The origin of *B. darwinianus*, an estuarine species, is intriguing. One plausible scenario involves a shallow sea with variable conditions of salinity, from normal to low (Pérez et al. 2011), known as the “Paranaense” or “Entrerriense” Sea, that covered a wide area of Argentina, Uruguay, and southern Brazil during the Middle Miocene (Martínez and del Río 2002), giving the conditions for their speciation. However, there are no related fossil forms in the deposits left by the Paranaense Sea. The only fossil material is associated with *B. rodriguezii* (del Río 1991). This fact leads us to think about the possible role of the connection between the Atlantic and the Caribbean Sea during the Caribbean and Paranaense marine transgressions of the Miocene, as has been suggested for other invertebrate groups (e.g., tube anemones, Stampar et al. 2012), although the existence of such connection has been questioned (Hernández et al. 2005; Wesselingh and Salo 2006; Cooke et al. 2012). Assuming that there was a connection, this seaway could have allowed the ancestral species, adapted to tolerate very low salinities and shallow estuarine environments, to enter to the southwestern Atlantic. We speculate that, following the closure of the trans‐Amazonian seaway, the population limited to the Caribbean Sea likely became extinct while the other population became restricted to the southwestern Atlantic. The origin of *B. darwinianus* remains a puzzle that is beyond the scope of the present study.

Finally, as indicated by Trovant et al. (2013), *B. rodriguezii* is a distinct species that diverged very early in the history of the genus. The presence of related fossil forms in the Late Miocene of the Paranaense province motivated the hypothesis of a long evolutionary history of this species in this region.

### Genetic diversity, population structure, and demographic history

The three species distributed in the warm‐temperate region of the southwestern Atlantic, *B. solisianus*,* B. darwinianus,* and *B. rodriguezii*, differed in their population structure and genetic diversity as well as in their demographic history. *Brachidontes solisianus* exhibited signs of a recent population expansion and, despite its wide distribution along the Brazilian coast, showed no genetic differentiation among populations. *Brachidontes darwinianus* and *B. rodriguezii* showed instead significant genetic differentiation among populations, without evidence of recent changes in population size. The population structure observed among *B. darwinianus* populations may be explained by the fact that this species is typical of estuarine environments, which are often discontinuous and separated by extensive marine shoreline potentially acting as barriers to dispersal. *Brachidontes rodriguezii* also exhibited differences among some of the populations. The population from Santa Clara del Mar in Argentina (Fig. 1), for instance, differed from populations in more southern locations, a result that can be explained by the hundreds of kilometers of coastline with sandy beaches and muddy tidal flats (SEGEMAR 2000) that separate these locations, habitats which are unsuitable for small mussels.

To synthesize, four species of intertidal scorched mussels exist along the Atlantic coast of South America. Their similar physiognomy belies their vastly divergent origin. *Perumytilus purpuratus,* the southernmost species and the only scorched mussel that is found in the cold‐temperate waters of southern South America (i.e., southward of 41°S), is also found in the Pacific Ocean along the coast of Chile. This species is closely related to *Austromytilus rostratus* from Australia and has thus a Gondwanan origin with its presence in South America linked to vicariance and dispersal (Trovant et al. 2015). Northward of the North Patagonian gulfs from approximately 43^o^S (Chubut, Argentina), the second species *Brachidontes rodriguezii* first coexists and then replaces *P. purpuratus* and is found up to Garopaba (28.9°S, Brazil). This is the only species thought on the basis of its phenotypic resemblance with local fossils to have regional ancestry dating back to the Miocene (del Río 1991). The third species, *Brachidontes darwinianus,* an estuarine species, is found from Punta del Este (Uruguay) to Bahia (Brazil). This species appears to be unrelated to any of the species we examined (Figs. 2, 3, 4). One plausible hypothesis is that an ancestor of *B. darwinianus* may have reached the estuaries of the southwestern Atlantic via the Trans‐Amazonian seaway that existed during the last marine transgression. The fourth species, *Brachidontes solisianus* is distributed along the coast of Brazil from Ceará to Rio Grande do Sul and its closest relative is *B. exustus I sensu* Lee and Ó Foighil (2005) found in the Caribbean northwest of the plume formed by the Amazon and Orinoco rivers, believed to have been the barriers responsible for its diversification. It is thus safe to conclude that despite the very similar phenotypic appearance of the mussel beds of the southwestern Atlantic, the presence and distribution of the four species in this region are the result of a complex suite, a collage, of diverse historical and ecological processes acting at different times.

## Conflict of Interest

None declared.

## Data Accessibility

DNA sequences: Genbank accessions number for 18S sequences are KT192105‐KT192084, for 28S sequences are KT192106‐192130 and for COI sequences are KT318184‐KT318264.

## Supporting information


**Data S1.** Model Selection.
**Table S1.1** Bayes Factor (BF) calculations based on HME for the different combinations of models, and for the three genes (COI and 18S‐28S).
**Table S1.2** Comparison of substitution and tree models, following the AICM approach; mitochondrial and nuclear datasets.
**Data S2.** Geographic and ecologic distribution of *Brachidontes* spp.
**Data S3.** Revised museum material.
**Table S3.1** Collection sites of the *Brachidontes* samples deposited in the “Museo Oceanográfico Eliezer Ríos” (MOFURG) and the “Museo de Historia Natural de Montevideo” (MHNM).
**Data S4.** Phenotypic (shell) characters examined in species considered in this study.
**Table S4.1** Phenotypic characters of *Brachidontes* spp.
