# Supplementary material for: Scorched mussels (Brachidontes spp., Bivalvia: Mytilidae) from the tropical and warm‐temperate southwestern Atlantic: the role of the Amazon River in their speciation
Source: Ecol Evol. 2016 Feb 18;6(6):1778–98. doi: 10.1002/ece3.2016 (PMC4758806; doi:10.1002/ece3.2016)
Supplement: Supplementary file 1 — Data S1. Model Selection. Table S1.1 Bayes Factor (BF) calculations based on HME for the different combinations of models, and for the three genes (COI and 18S‐28S). Table S1.2 Comparison of substitution and tree models, following the AICM approach; mitochondrial and nuclear datasets. Data S2. Geographic and ecologic distribution of Brachidontes spp. Data S3. Revised museum material. Table S3.1 Collection sites of the Brachidontes samples deposited in the “Museo Oceanográfico Eliezer Ríos” (MOFURG) and the “Museo de Historia Natural de Montevideo” (MHNM). Data S4. Phenotypic (shell) characters examined in species considered in this study. Table S4.1 Phenotypic characters of Brachidontes spp. [file ECE3-6-1778-s001.doc]

**SUPPORTING INFORMATION**

The Supporting Information (S) presented below consists of a series of self-contained components providing data relevant as support for the study. They are numbered consecutively in the order in which they are referred to in the text.

**Data S1.** Model Selection.…………..………………………………………..…………….……1

**Data S2.** Geographic and ecologic distribution of *Brachidontes* spp…………….………...........3

**Data S3.** Revised Museum Material………………………………..……………….……...........4

**Data S4.** Phenotypic (shell) characters examined in species considered in this study...…….....14

**Data S1. Model Selection**

The General Time Reversible model (GTR+G+I) was selected for ML estimation with the nuclear and mitochondrial dataset based on the AIC criterion. For the 28S rDNA nuclear dataset the AIC weight of the selected model was 0.9637. Comparing the first two models of substitutions in the rank generated by jModeltest (GTR+I+G and GTR+G) with the decision table of Kass & Rafftery (1995), we obtained decisive evidence against GTR+G (ΔAIC=8.84). For the 18S rDNA nuclear dataset the AIC weight of the selected model was 0.2799. Comparing the first two models of substitutions in the rank generated by jModeltest (GTR+I+G and GTR+I) with the decision table of Kass & Rafftery (1995), we obtained substantial evidence against GTR+I (ΔAIC=0.9441). For the mitochondrial dataset, the AIC weight of the selected model was 0.99. Comparing the first two models of substitutions in the rank (GTR+I+G and TIM3+I+G) we obtained a ΔAIC of 9.49, implying decisive evidence against TIM3+I+G.

Phylogenies reconstructed with Bayesian Inference (BI) were estimated with different substitution (HKY+G+I, Hasegawa *et al.* 1985, and GTR+G+I, Tavaré, 1985) and tree (Yule and Birth-Death processes) models. The marginal-likelihood scores of the posterior distributions were compared using the decimal logarithm of Bayes Factors (BFs, Kass & Raftery, 1995), defined as

Log10 [Pr (D | H1)] – Log10 [Pr (D | Ho)].

Two different methods were applied: harmonic mean estimation (HME, Newton & Raftery, 1994) and a posterior simulation-based analog of the Akaike information criterion through Markov chain Monte Carlo analysis (AICM, Raftery *et al.* 2007). The substitution and tree models favored by BFs were the GTR+I+G and birth-death models for the three genes, whether the HME (Table S1.1) or AICM (Table S1.2) method was followed.

**Table S1.1** Bayes Factor (BF) calculations based on HME for the different combinations of models, and for the three genes (COI and 18S-28S). Values from 0 to 0.5, indicate that there is not evidence against Ho; 0.5 to 1, substantial evidence against Ho; 1 to 2, strong evidence against Ho and higher than 2 indicate decisive evidence against Ho. Also, a higher probability value indicates better model fit (indicated in bold).

|  | **Substitution Model** | |  |
| --- | --- | --- | --- |
| **Tree Model** | H_1_: GTR+G+I | Ho: HKY+G+I | **BF** |
| **COI** H_1_: Yule | -10024.24 | -10053,13 | 28,89 |
| Ho: Birth-Death | -**9902.296** | -9920,513 | 18,217 |
| **BF** | 121,944 | 132,617 |  |
| **28S** H_1_: Yule | -3487.471 | -3491.369 | 3.898 |
| Ho: Birth-Death | **-3412.697** | -3451.629 | 38.95 |
| **BF** | 74.774 | 39.74 |  |
| **18S** H_1_: Yule | -3939.393 | -3946.221 | 6.828 |
| Ho: Birth-Death | **-3920.999** | -3927.848 | 6.849 |
| **BF** | 18.394 | 18.373 |  |

**Table S1.2** Comparison of substitution and tree models, following the AICM approach; mitochondrial and nuclear datasets. A lower AIC value (in boldface in the table) indicates better model fit.

|  | **Substitution Model** | |
| --- | --- | --- |
| **Tree Model** | GTR+G+I | HKY+G+I |
| **COI** Yule | 20412,319 | 20441,811 |
| Birth-Death | **19952,451** | 19988,278 |
| **28S** Yule | 7076.573 | 7089.185 |
| Birth-Death | **6867.544** | 6942.468 |
| **18S** Yule | 7918.654 | 7928.766 |
| Birth-Death | **7867.741** | 7878.654 |

**Data S2. Geographic and ecologic distribution of *Brachidontes* spp.**

*Brachidontes solisianus* was originally described by d´Orbigny (1842, 1846) based on samples collected at Maldonado (Uruguay) and Rio de Janeiro (Brazil), although the British Museum labels indicate only the first of these two locations. Since its original description, *B. solisianus* was not cited again for Uruguayan waters. As part of this study we surveyed the area covered by d´Orbigny guided by his travel diary. He observed "*sur les roches des environs de Maldonado, a l'embouchure de la Plata. Elle s'y milliers attache sur les roches par*". On the site where presumably he made his observations (Punta Ballena) only *B. rodriguezii* was found in great abundance and *B. darwinianus* in lower abundance and circumscribed to some pools. Two hypotheses may explain the absence of this species in the area. The first is that in the time that d´Orbigny explored the Uruguayan coast this species was present in the area, but has disappeared from there since. The second is that what he observed was actually *B. rodriguezii*. In this case the material deposited in the British Museum should have been collected in Rio de Janeiro, not Maldonado as indicated in the labels. The answer to this problem is beyond the scope of this study.

There are, however, numerous records of *B. solisianus* for the Brazilian coast north of Santa Catarina (28° S). As part of this study we also visited the few hard substrate habitats present on the coast of Rio Grande do Sul, characterized by approximately 680 km of sandy beaches extending from La Coronilla (Uruguay) and Cabo Torres (North of Rio Grande do Sul). The only interruptions are the docks of Rio Grande (31.5° S), built in 1915, and the hull of the ship Altair, stranded in the intertidal of Praia do Cassino (32° S) in the early 1970s. In the latter, a few isolated specimens of *B. solisianus* were collected, constituting validated southernmost record of the species. The study of materials deposited at the Museum of Rio Grande (Table S5.1) broadened its northern edge distribution to 3° S, in the locality of Praia do Futuro (Ceará, Brazil).

*Brachidontes rodriguezii* (d´Orbigny, 1842) is usually reported from the docks of Rio Grande do Sul (32° S) to the north Patagonian gulfs (41°- 43° S) (Ríos, 1994), supported by the sequenced specimens. However, the northern limit of the distribution of *B. rodriguezii* according to the revised material of museums (Table S5.1) indicates that extends to Garopaba (Santa Catarina, Brazil, 28° S).

The presence of *Brachidontes darwinianus*, an estuarine species, is well documented for the Uruguayan coast and in eastern and southern Brazil, which overlap with *B. solisianus* in transitional habitats. The southern limit of the distribution of *B. darwinianus* is not well established. d´Orbigny (1842) original description based on material collected in Bahía Rosas (Río Negro Province, Argentina), Montevideo (Uruguay) and Río de Janeiro (Brazil). The material collected as part of this study in Bahía Rosas was sequenced and corresponds to *B. rodriguezii*. Despite a careful search, *B. darwinianus* was not observed in the area. There may be has been confusion with juvenils of *B. rodriguezii* which exhibit a phenotype similar to *B. darwinianus* (Trovant *et al.* 2013). The material studied (own and museum collections, table S5.1) extends the range of distribution of this species from Montevideo (34° S, Uruguay) to Bahía (13° S, Brazil).

**Data S3. Revised Museum Material**

**Table S3.1** Collection sites of the *Brachidontes* samples deposited in the "Museo Oceanográfico Eliezer Ríos" (MOFURG) and the " Museo de Historia Natural de Montevideo " (MHNM).

| **Museum** | **Repository** | **Locality** | **State** | **Country** | **Collector-& date** | **Latitude South** | **Longitude West** | **Specimens** | **Observations** |
| --- | --- | --- | --- | --- | --- | --- | --- | --- | --- |
| ***B. rodriguezii*** | | | | | | | | | |
| MHNM | 1614 | Aguas Dulces | Rocha | Uruguay | E. gerzenstein, Feb. 16, 1964 | -34.27 | -53.77 | 1 large |  |
| MOFURG | 5.170^1^, 12.756^2^ | Albardão | Río Grande do Sul | Brazil | M. Oliveira leg. Mar., 1959^1^, Nov., 1967^2^ | -30.26 | -50.22 | Many, assorted sizes^1^, several, relatively small^2^ | 12 milhas SE Albardão, Pescal II^1^, 15-18 m, s/âncora. “Milicent King” ^2^ |
| MHNM | 1537^1^, 1541^2^, 1515^3^ | Bahia Blanca | Buenos Aires | Argentina | Fontana^1^, Bicego, 1892^3^ | -38,9 | -62,09 | 7^1^, 3^2^, 6^3^ | Puerto Militar^1-2^, Lote 11121 Museo Arg. C. Nat. “Bernardino Rivadavia” ^2^ |
| MHNM | 1595 | Balneario Bella Vista | Canelones | Uruguay | M.A. Klappenbach, Jun. 17, 1962 | -34,8 | -55,34 | 10 |  |
| MHNM | 1523 | Boca del Río Negro | Río Negro | Argentina | Berg, Set., 1874 | -41,04 | -62,78 | Several |  |
| MHNM | 1587^1^, 1659^2^, without ID^3^, 1585^4^ Without ID^5^ | Cabo Polonio | Rocha | Uruguay | M.A. Klappenbach col, 11/Marzo/1958^1^ M.A. Klappenbach Marzo 1961^2^ F. Achaval 16-01-1988^3^ M.A. Klappenbach^4^ M.A. Klappenbach Feb/1955^5^ | -34.4 | -53.79 | Very large^1^, 6 large^2^, 1^3^, numerous, mostly large^4^, several, mostly large^5^ | En barca de pescadores^3^ |
| MHNM |  | Santa Maria de Rocha | Rocha | Uruguay | O.E.S. Sicardi, Jun. 21, 1980 | -34.66 | -54,21 | 4 | CR.236 |
| MHNM | 1519 | Carmen de Patagones | Río Negro | Argentina | Bicego | -41,03 | -62,79 | Several |  |
| MHNM ^1-4^ – MOFURG^5-6^ | 8270^1^, 899^2^, 11337^3^, 7617^4^,  11.187^5^, 40.202^6^ | Chui | Río Grande do Sul | Brazil | Barcellos B.N.^2^, Olazarri y F. Achaval, Jul. 20, 1972^3^, Jul. 22, 1972^4^, E. Martino, Feb. 1, 1966^5^, José Carlos Tarasconi ^6^ | -33.74 | -53.36 | Several, some large^1^, 1^2^, 5 assorted sizes^3^, , many, assorted sizes^4^, Many^5^, 3 complete shells^6^ | Campaña Hero^1-4^prof. 10m, roca y conchilla^1^, 20m prof, Fondo de barro^3^, 10m, barro y piedra, estación 3A^4^. 33° 50’S, 53° 25’W^1^, 34° 00’S, 53° 28’W^3^, 33° 47’S, 53° 23’W^4^. Attached to rocks, intertidal^6^ |
| MHNM | 1606^1^, 1612^2^, | Isla de Lobos | Maldonado | Uruguay | Eduardo Grimber, Jun. 16, 1962^1^, M.A. Klappenbach, Feb., 1955^2^, | -35,02 | -54,88 | 1 small^1^, 8^2^, | Trabajo de buzo, 15m prof., Entre el Islote y la Isla de Lobos^1^, |
| MOFURG | 23.660-23.661 | Garopaba | Santa Catarina | Brazil | Felipe Dias, Apr., 1970 | -28.02 | -48.61 | several | labeled as *Brachidontes/Ostrea,* 3 m |
| MHNM | 1597 | Isla Gorriti | Maldonado | Uruguay | Dr. F. de Buen | -34,95 | -54,96 | several |  |
| MHNM |  | José Ignacio | Maldonado | Uruguay | Sicardi | -34,84 | -54,63 | 5 |  |
| MHNM^1-5^, MOFURG^6-7^ | 284^1^, 1602^2^, 1647^3^, 1586^4^, 7726^5^, 19.065^6^, 16.246^7^ | La Coronilla | Rocha | Uruguay | Marzo 1970^1^, Pablo R. San Martin Dec. 16, 1963^2^, F. Mañé Garzón, Feb.,1963^3^, M.A. Klappenbach, Dec. 4, 1964^4^, “Hero”1972-3A. Jul. 19, 1972^5^, Linda Rios, Feb., 1977^6^, Nov.1971^7^ | -33.89 | -53.5 | Several, assorted sizes^1^, 1^2^, several, assorted sizes^3^, Several^4^, 1^5^, Several^6^, Several^7^ | Est. 2. Al Norte de Isla Verde, a 1 Milla de La costa. Prof: 8m. 33° 56’S, 53° 28’W,Fondo: conchilla^5^, Turma malacologia^7^ |
| MHNM^1-12^ MOFURG^13^ | 1594^1^, 1653^2^, 1591^3^, 1588^4^, 4490^5^, 1604^6^, 1630^7^, 1582^8^, 1590^9^, without ID^10^, without ID^11^, without ID^12^, 19.059^13^ | La Paloma | Rocha | Uruguay | M.A. Klappenbach, Mar., 1966^1^, Adolfo Pose, Feb. 2, 1964^2^, M.A. Klappenbach, Jan. 12, 1957^3^, M.A. Klappenbach & Gardiol^4^, Ravera A., Abr. 28, 1967^5^, Adolfo Pose, Jun., 1963^6^, F. de Buen, Apr. 18, 1950^7^, M.A. Klappenbach, Oct. 4, 1959^8^, Adolfo Pos, Aug. 26, 1962^9^, Sicardi, Feb. 18, 1961^10^, Sicardi, Set. 29, 1963^11^, Sicardi, Set. 29, 1963^12^, Mario Cachés, Dec. 1973^13^ | -34.65 | -54.15 | Many, assorted sizes^1^, 1 shell and 1 valve^2^, several, mostly large^3^, 5 one exceptionally large, the largest measure 58.3mm^4^, 6^5^, 7 valves^6^, 1^7^, 3^8^, many^9^, 8^10^, 4^11^, 3^12^, 1^13^, | “Sagitário/ Pesca Blanca^5^, Cabo Santa María^12^, |
| MHNM | 1581^1^, 1634^2^ | La Pedrera | Rocha | Uruguay | José Olazarri, Jun. 3, 1963^1^, Eliseo Duarte, Oct. 22, 1963^2^ | -34.59 | -54.12 | 7^1^, many^2^ |  |
| MOFURG | 10.331^1^, 9.039^2^ | Mar del Plata | Buenos Aires | Argentina |  | -37,99 | -57,54 | 4 shells^1^, 7 complete shells^2^ |  |
| MOFURG | 32.832^1^, 23.989^2^ | Rio Grande | Rio Grande do sul | Brazil | Jesus, Nov. 8, 1992^1^, Capítoli-Berê, Nov. 1985^2^ | -32.16 | -52.08 | 8 large + 2 small complete shells^1^, Juvenils^2^ | Molhe Leste, Barra de Rio Grande^1^, Molhe Oeste Barra de Rio Grande^2^ |
| MHNM | 1540 | Necochea | Buenos Aires | Argentina | Bicego, 1897 | -38,58 | -58,71 | 3 |  |
| MHNM^1^, MOFURG^2^ | 0070^1^, 35.761^2^, | Piriapolis | Maldonado | Uruguay | Eliseo Duarte, Mar. 1959^2^ | -34,87 | -55,27 | 1^1^, 2 complete shells, very large^2^ | Antigua Colec. Mus. Nac. Hist. Nat. N°201^1^, Punta Negra^2^ |
| MHNM | 10.274 | Playa de Santa Teresa | Rocha | Uruguay | Susana Maytia, Feb., 1974 | -34 | -53.53 | 2 |  |
| MHNM |  | Puerto Pirámides | Chubut | Argentina | Mar. 31, 1979 | -42.58 | -64.28 | 1 |  |
| MHNM | 1637 | Punta Ballena | Maldonado | Uruguay | Dr. Elias Ureta | -34,89 | -55,04 | 8 |  |
| MHNM^1-12^, MOFURG^13^ | 1609^1^, 1598^2^, 2523^3^, 1649^4^, 1633^5^, 1584^6^, 1658^7^, 1589^8^, 14278^9^, 1593^10^, without ID^11^, without ID^12^, 35.735^13^ | Punta del Este | Maldonado | Uruguay | J.I. Muñoa, Dec. 27, 1956^1^, M.A. Klappenbach, Jun. 16, 1962^2^, E. Duarte col, Mar., 1957^3^, M.A. Klappenbach, Jun. 16, 1962^4^, M.A. Klappenbach, Feb., 1955^5^, Ema y Esther Morales^6^, Eliseo Duarte, Mar., 1958^7^, M.A. Klappenbach 1962^8^, E.H. Ureta, May. 1, 1978^9^, M.A. Klappenbach, Aug., 1962^10^, Sicardi, Jul. 8, 1960^11^, Sicardi, Set. 17, 1961^12^, Eliseo Duarte, Mar. 1957^13^ | -34,96 | -54,94 | 1^1^, 2^2^, 5^3^, 1^4^, 8^5^, 2^6^, Several^7^, several, mostly small^8^, 1 + 1 valve, the largest one 56.9mm^9^, several^10^, 2 large, 51.1mm^11^, 4^12^, 6 complete, medium size^13^, | Playa Mansa^2^, Playa Mansa^4^, Playa Mansa^8^, Playa las Delicias^9^, Playa La Pastora^11^, Playa La Pastora^12^, Mariskonea, labeled *Brachidontes solisianus*^13^, |
| MOFURG | 8.413 | Punta Médanos | Buenos Aires | Argentina | Dec., 1962 | -39,44 | -62,02 | Several | 27 m, fundo rochoso. Pescal 2. |
| MHNM | 7505 | Punta Villarino, Valizas San Matías, Golfo San Matías | Río Negro | Argentina | V. Scarabino y S. Maytia, Feb. ,1972 | -40,82 | -64,89 | 6 | Litoral |
| MHNM^1^, MOFURG^2-6^ | 1638^1^, 10.478^2^, 2.949^3^, 2.232^4^, 5.968^5^, 4.026^6^ | Sarita | Rio Grande do Sul | Brazil | Rios E.C, Dec., 1957^1^, E. Rios, Mar. 4, 1964^2^, E.Rios^3^, E.C. Rios^4^, E.C.Rios, Feb. 7, 1960^5^, E.C.Rios, Mar., 1957^6^ | -32.66 | -52.43 | Several^1^, Many^2^, 1 complete shell^3^, 2^4^, 5 shells^5^, 3 complete shells^6^ | Batelâo, Lat: Batelâo, Long: B.51^1^, Batelão B51^2^, s/o Batelão B51^5^, sôbre o batelão afundado^6^ |
| MHNM | 1603 | Torres | Rio Grande do Sul | Brazil | Thomé J.W., 13-Jul. 15, 1957 | -29.33 | -49.71 | 3 |  |
| MOFURG | 18.799 | Tramandaí | Rio Grande do Sul | Brazil | Merg, Jan., 1976 | -29.98 | -50.12 | 2 right valves | Labelled as *Brachidontes exustus*. Boia terminal. |
| MHNM | 3203^1^, 3455^2^, 1622^3^, 3514^4^, 7730^5^, 7735^6^ | Valizas | Rocha | Uruguay | “Hero”Est. 13, 21 de Julio de 1972^1^,, Olazarri col. 23 Jul. 1972^2^, F de Buen, Marzo 1949^3^, Olazarri, 21/Jul/1972^4^, “Hero” 1972, 21/Jul/1972^5^, “Hero” 1972-3A, 23 Jul. 1972^6^, | -34.33 | -53.78 | Numerous, assorted sizes^1^, 12, mostly small ^2^, 1 + 1 valve^3^, numerous^4^, 12 small^5^, many small^6^ | Entre Valizas y Punta Palmar. 2 ½ millas de la costa Est. N°22 “Hero” Prof. 12 m, Conchilla^2^, Frente a Valizas a 1.50 millas de La costa, Prof.14m, Conchilla est. “Hero” ^4^, Est. 13, frente a Valizas, a 2 millas de la costa, prof: 15m, fondo: conchilla y roca^5^, Est. 22, entre Valizas y Punta Palmar, a 2.5 millas de la costa, , prof: 12m, fondo: conchilla^6^ |
| MHNM | 1542 | Bahía Camarones | Chubut | Argentina | Bicego, 189 | -44,8 | -65,71 | 3 |  |
| ***B. solisianus*** | | | | | | | | | |
| MOFURG^1^, MHNM^2^ | 12.731^1^, 0915^2^ | Praia do Futuro | Ceará | Brazil | H.R. Matthews, Feb., 1965^1^, Dec. 16, 1956^2-^ | -3,72 | -38,45 | Several small^1^, Several^2-^ | Intertidal, surf zone^1^, Itapebussu, Inst. Ocean. de S. Paulo^2^. |
| MOFURG | 16.709 | Itamaracá | Pernambuco | Brazil | R.L. Melo, Summer,1972 | -7,74 | -34,82 | Many | S/pedra |
| MHNM | 0870^1^, 1506^2^ | Recife | Pernambuco | Brazil | Jul. 27, 1962^1^, Jul. 27, 1962^2^ | -8,08 | -34,87 | Numerous^1^, Many^2^ | Praia da Piedade. N°4 Instituto Oceanográfico de Recife^1^, Praia da Piedade. N°2 Instituto Oceanográfico de Recife^2^ |
| MHNM^1^, MOFURG^2-3^ | 0933^1^, 1.138^2^, 12.750^3^ | Paripueira | Alagoas | Brazil | Pablo de Sa Cardoso^1^, P.S. Cardoso^2^, Sá Cardoso, 1967^3^ | -9,46 | -35,54 | 3^1^, Several^2^, Several^3^ | S/arenito^3^ |
| MOFURG | 36.889 | Maceió | Alagoas | Brazil |  | -9,66 | -35,69 | Several | Ponta Verde |
| MOFURG | 11.282^1^, 16.603^2^ | Salvador de Bahia | Bahia | Brazil | Saulo G. Paes, Jul. 31, 1966^1^, Newton Salles, 1970^2^ | -12,95 | -38,34 | Many^1^, Many^2^ | Praia Farol, Itapoan. M/baixa, s/rochas^1^, Pituba^2^ |
| MOFURG | 24.328 | Guarapari | Espiritu Santo | Brazil | P. Young, Jul., 1984 | -20,67 | -40,49 | 5 small shells | Labeled as *Brachidontes* sp. A/rochas |
| MOFURG | 5.071^1^, 20.479^2^, 19.879^3^ | Cabo Frio | Rio de Janeiro | Brazil | E. Rios, Dec. 27, 1958^1^, BHS, Jun., 1972^2^, BHS, Jun., 1972^3^ | -22,89 | -42,02 | 2 shells^1^, several small complete shells^2^, several^3^ | Labeled as *Brachyodontes exustus*. P. Forte^1^, P. Forte^2^, Tayo^3^ |
| MOFURG | 36.908 | Mangaratiba | Rio de Janeiro | Brazil | E. Rios | -22,96 | -44,04 | Several, some relatively large |  |
| MOFURG | 5.062 | Arraial do Cabo | Rio de Janeiro | Brazil | E. Rios, Dec. 26, 1958 | -22,97 | -42,01 | Numerous | One of two labels read *~~exustus~~ solisianus* |
| MHNM | 1744^1^, 1596^2^, 1636^3^ | Ubatuba | San Pablo | Brazil | Plinio S. Moreira, Aug. 12, 1956^1^, M.A. Klappenbach, Aug. 14, 1959^2^, M.A. Klappenbach, Feb. 20, 1962^3^ | -23,46 | -45,05 | 2^1^, 5^2^, 2^3^ | Ensenada do Flamengo^1^, Praia do Tenório, sobre piedras^3^ |
| MHNM | 1499^1^, 0896^2^ | San Sebastián | San Pablo | Brazil | Barón de Fiore^1^, Sergio Rodriguez & José Olazarri, Jul. 20, 1962^2^ | -23,8 | -45,39 | 9^1^, 3^2^ |  |
| MHNM | 1498 | Santos | San Pablo | Brazil | Barón de Fiore | -23,97 | -46,32 | 5 |  |
| MHNM | 1495 | Cananeia | San Pablo | Brazil | Liliana [Forneri F. Scarabino com. Pers], Oct., 1956 | -25,04 | -47,88 | Many | Punta Itacurussá |
| MOFURG | 5.183 | Paranagua | Parana | Brazil | Flavio, Jul., 1952 | -25,45 | -48,39 | 6 shells | S/rochas, lavagem de ostras. |
| MOFURG | 5.214 | Ilha do Mel | Parana | Brazil | Morretes, Set., 1952 | -25,52 | -48,33 | Many |  |
| MOFURG | 20.064 | Piraquara | Paraná | Brazil | P. Costa, Set., 1971 | -25,6 | -48,36 | Several | Near Angra dos Reis, RJ |
| MOFURG | 11.733 | Matinhos | Parana | Brazil | F. Zanardini, 1960 | -25,83 | -48,53 | 10 shells |  |
| MHNM | 3131^1^, 5.221^2^, without ID ^3^ | Caioba | Parana | Brazil | Lange R., Dec. 11, 1954^1^, Dec. 11, 1954^2^, F. Zanardini, Apr. 30, 1959^3^ | -25,85 | -48,55 | 4^1^, 1^2^, Numerous^3^ | Ilha Rochas^3^ |
| MOFURG | 11.737^1^, 11.738^2^ | Guaratuba | Parana | Brazil | F. Zanardini, 1962^1^, F. Zanardini, Jan., 1963^2^ | -25,88 | -48,56 | Many^1^, Numerous, some relatively large^2^ | Ilha do Saí^2^ |
| MOFURG | 23.166 | Itapema | Santa Catarina | Brazil | E. Rios & C. Somini, Mar. 1985 | -27,09 | -48,61 | 3 | Labeled as *Brachidontes* sp. s/rochas |
| MOFURG | 42.940^1^, 45.122^2^ | Bombinhas | Santa Catarina | Brazil | JCT, Feb., 1998^1^, JCT, Dec., 1998^2^ | -27,14 | -48,49 | 6 shells + a left valve^1^, 4 complete shells^2^ | Aderidas a pedras, intertidal^1^, Praia do Canto Grande. Sobre pedras, supratidal^2^ |
| MOFURG | 20.032 | Porto Belo | Santa Catarina | Brazil | Thierry Rios, Feb. 1978 | -27,15 | -48,54 | Several | S/rochas |
| MOFURG | 37.517 | Ilha de Santa Catarina | Santa Catarina | Brazil |  | -27,6 | -48,43 | 6 complete shells |  |
| MOFURG | 31.486 | Florianópolis | Santa Catarina | Brazil | Flávio Cavalhi, Jan., 1978 | -27,69 | -48,48 | 9 shells | Labeled as *Brachidontes exustus*. Ilha do Campeche |
| MHNM ^1-2^, MOFURG^-3^ | 0883^1^, 0892^2^, 1.499^3^ | Torres | Rio Grande do Sul | Brazil | Erika H. Buckup, Set. 9, 1956^1^, J.W. Thomé, Jul. 13-15, 1957^2^, E. Rios, Oct., 1956^3^ | -29,33 | -49,71 | 2^1^, Several large^2^, Several^3^ | Muelle del Puerto^1^, N° 526 Mus. Rio Gr. Cienc. Nat. Porto Alegre^2^, s/rochas^3^ |
| ***B. darwinianus*** | | | | | | | | | |
| MOFURG | 374^1^, 34.392^2^,  11.527^3^, 12.320^4^ | Salvador de Bahía | Bahia | Brazil | H.S. Lopes^1^, S. Paes, Oct. 1966^3^, Saulo & Rios, Jul. 16, 1967^4^ | -13 | -38.53 | 2^1^, l large shell, very neat stration^2^, 8 shells + a left valve^3^, 12 shells^4^ | Labeled as *Brachydontes exustus.* Peninsula de Itapagipe^1^, Labeled as *Brachidontes (Hormomya) exustus.* Peninsula de Itapagipe^2^, Labeled as *Brachidontes exustus*, s/rocha. Montserrat^3^, Labeled as *Brachidontes exustus*, s/rochas, Ilha de Maré^4^ |
| MOFURG | 51.858 | Sao João da Barra | Rio de Janeiro | Brazil | P.S.Oliveira, Jul. 27, 2012 | -21,64 | -41,01 | 4 single valves, one long and narrow | Labelled as *Brachidontes solisianus.* Praia de Chapéu do Sol. |
| MOFURG | 19.881 | Cabo Frio | Rio de Janeiro | Brazil | Marise, May, 1972 | -22.71 | -42.04 | 2 shells | Labeled as *Brachidontes exustus* |
| MOFURG | 36.906 | Mangaratiba | Rio de Janeiro | Brazil | E Rio | -22.96 | -44.04 | 6 complete shells |  |
| MOFURG | 20.929^1^, 85^2^ | Sepetiba | Rio de Janeiro | Brazil | LCA, 1972^1^, E. Rio and H.S. Lopes^2^ | -22,97 | -43,7 | 4 complete shells^1^, 3 complete shells^2^ | Na lama sobre pedras^1^, Dois Rios, Recreio dos Bandeirantes, Ilha Grande and B. Sepetiba^2^ |
| MHNM^1^, MOFURG^2^ | 0953^1^, 36.897^2^ | Isla Grande | Río de Janeiro | Brazil | H de Souza Lopes^1^, E Rio. H.S. Lopes leg.^2^ | -23,1 | -44,25 | 2^1^, 1 complete shell^2^ | Bahia Sepetib. Inst. Ocean. De S. Pablo, Brasil^1^, Praia Dois Rios^2^ |
| MOFURG | 12.515 | Ubatuba | San Pablo | Brazil | Carlos Ozores | -23,45 | -45,05 | 3 shells + 3 valves | Labeled as *Brachidontes exustus.* Claro River and Dura Beach. |
| MOFURG | 17.251 | Boraceia | San Pablo | Brazil | L.A.F. | -23,75 | -45,81 | 2 complete shells | Sobre rochas a beira-mar |
| MOFURG | 11.267 | Bertioga | San Pablo | Brazil | L.A.F., Oct. 16, 1971 | -23,8 | -46,02 | 3 juvenils | Labeled as *Brachidontes exustus*. Sôbre rochas a beira mar |
| MHNM | 1504 | San Sebastián | San pablo | Brazil |  | -23,8 | -45,39 | 1 |  |
| MHNM | 1501 | Santos | San pablo | Brazil |  | -23,97 | -46,32 | 6 | Entre ostras, Colección Ihering |
| MHNM | 1503^1^, 1500^2^ | Isla Comprida | San Pablo | Brazil | Baron de Fiore^1^, Krone R.^2^, | -24,74 | -47,54 | 3 valves^1^, 1^2^ | Iguape^1-2^, |
| MHNM | 898 | Cananeia | San Pablo | Brazil | Baron de Fiore | -25,04 | -47,88 | 1 | Colección de Fiore Inst. Ocean. São Paulo |
| MOFURG | 11.736 | Caiobá | Paraná | Brazil | Lange de Morretes, Jun. 16, 1953 | -25,85 | -48,55 | 5 complete shells + a left valve | Rochas |
| MOFURG | 36.899 | Guaratuba | Paraná | Brazil |  | -25,88 | -48,56 | 3 complete shells |  |
| MOFURG | 1.548 | Camboriú | Santa Catarina | Brazil | Feb. 5, 1954 | -27 | -48,6 | 2 complete shells |  |
| MOFURG | 47.813^1^, 42.876^2^ | Porto Belo | Santa Catarina | Brazil | JCT, Aug. 17, 2002^2^ | -27,15 | -48,57 | 1^1^, several loose valves^2^, | Labeled as *Brachidontes exustus.* Praia do Pereque. Vazia na praia^1^, labeled as *Brachidontes exustus*. Praia de Perequé. Jogadas a praia^2^ |
| MOFURG | 21.846^1^, 42.948^2^, | Florianopolis | Santa Catarina | Brazil | Gofferge, Oct. 1972^1^, JCT, Jul., 1997^2^ | -27,59 | -48,54 | Numerous, good size range^1^, 2^2^ | Lagoa, 1 m prof.  *~~darwinianus~~* crossed, annotated “¿?”^1^, Labeled as *Brachidontes exustus.* Aderidas a pedras, intertidal^2^ |
| MHNM^1^ MOFURG^2-4^ | 1746^1^, 15.214^2^, 16.827^3^, 6.863^4^ | Lagoa da Conceição | Santa Catarina | Brazil | Carvalho Rios, Feb., 1960^1^, Tremel, Jan., 1970^2^, Gofferjé^3^, E. Tremel, 1960^4^ | -27,61 | -48,47 | Several small^1^, many, clumps^2^, Several^3^ , many complete shells, relatively small^4^, | (Fundo: Rochas) ^1^, S / rochas, 0.5 m ^2^, Labeled as *Brachidontes exustus*^3^, 2 labels, one says *darwinianus*, the other *solisianus*; look like *darwinianus*^4^ |
| MOFURG | 4.618^1^, 4.618^2^ | Imbé | Rio Grande do Sul | Brazil | 1958^1^, E. Rios, 1958^2^ | -29,97 | -50,12 | 1 complete shell^1^, 8 complete shells, small^2^ | Barcos de pesca^1^, Imbé and Tramandaí^2^ |
| MOFURG | 21.618^1^, 22.712^2^, 18.947^3^ | Tramandaí | Rio Grande do Sul | Brazil | L.A. Pedroso, Apr. 2, 1981^1^, L.A. Pedroso, Apr. 2, 1981^2^, Vera, Inga e merguladores, Jan. 23, 1976^3^ | -29,98 | -50,12 | Several complete shells^1^, several complete shells^2^, 1 complete shell^3^ | Nas pedras^1^, Pedras. ^2^, Boia No 1 de Petrobrás^3^, |
| MHNM | 1653 | La Paloma | Rocha | Uruguay | Adolfo Pose, Feb. 2, 1964 | -34.65 | -54.15 | 5 valves |  |
| MHNM | Without ID | San Luis | Canelones | Uruguay | F. Scarabino, Jan., 2012. | -34,76 | -55,58 | 10 | Resaca, zona de “La Piscina” |
| MHNM | 1618 | Boca Arroyo Solis Chico | Canelones | Uruguay | Klappenbach, Mar. 1961 | -34,76 | -55,7 | Several |  |
| MHNM | 1667 | La Floresta | Canelones | Uruguay | Klappenbach, Mar. 2, 1963 | -34,76 | -55,67 | Many |  |
| MHNM | 1641 | Los Titanes | Canelones | Uruguay | Eliseo Duarte | -34,78 | -55,56 | 1 |  |
| MHNM | 1668 | Bella Vista | Canelones | Uruguay | Klappenbach, Jun. 17, 1962 | -34,8 | -55,34 | Many | Balneario |
| MHNM | 1665^1^, without ID^2^ | Las Flores | Maldonado | Uruguay | M.A. Klappenbach, Feb. 10, 1961^1^ | -34,81 | -55,33 | Many^1^, 1^2^ |  |
| MHNM | 1663^1^, without ID^2^ | Piriápolis | Maldonado | Uruguay |  | -34,87 | -55,27 | 11^1^, 2^2^ | Playa Grande. Resaca^2^ |
| MHNM | 1662^1^, 1530^2^, 1625^3^, 1655^4^, 1529^5^, 1620^6^, 1652^7^, 1650^8^, without ID^9^, without ID^10^ | Montevideo | Montevideo | Uruguay | Elias Ureta^1^, M.A. Klappenbach, Jan. 17, 1965^2^, Arturo Schlebinger & J. Eduardo García, Apr. 27, 1968^3^, C.S. Carbonell. Nov. 6, 1956^4^, Celta Klappenbach, Sep. 20, 1960^6^, Klappenbach^8^, Adolfo Pose ^9^, Sicardi, Oct., 1961^10^, M.A. Klappenbach, Sep. 15, 1957^1^, F. Scarabino, Apr. 30, 1995^2^ | -34,88 | -56,04 | 8^1^, 3^2^, Several^3^, Several^4^, Several^5^, 5 valves^6^, 1^7^, 2^8^, 1^9^, 5^10^ | Faro de Punta Carretas^1^, Punta Yeguas^2^, En Barco “Morena Clara”hundido a 12m de prof. frente a Escollera Sarandí^3^, Punta Gorda^4^, Puerto de Montevideo, en casco de Madera sumergido a 1.50m fondeado hace 8 años^5^, Playa Malvin^6^, Bahia de Montevideo^7^, Playa Buceo^8^, Rio de La Plata, pantanoso^9^, Playa Carrasco^10^, |
| MHNM | Without ID | Isla de Flores | Montevideo | Uruguay | Curso oceanografia FCIEN, May 27, 2008 | -34,94 | -55,92 | 6 | Rio de La Plata, 4.5m, draga, fondo de conchilla. Puerto |
| MHNM | 1666^1^, without ID^2^, 10825^3^ | Punta Del Este | Maldonado | Uruguay | M.A. Klappenbach, Aug.,1962^1^, M.A. Klappenbach, Aug., 1962^2^, Moyano M.R. ^3^ | -34,96 | -54,94 | Many^1^, 3^2^, 1 juvenil^3^ | Playa Mansa^1^, Playa Mansa (de Pta. Del Este a Montevideo) ^2^, apartado de 10825^3^ |

**Data S4. Phenotypic (shell) characters examined in species considered in this study**

The genus-level systematics and taxonomy of the Brachidontinae (Mytilidae) is based on phenotypic characters observable in the shell. Here we present details of the conchological characters of the materials used for sequencing. This information may prove valuable in future studies linking molecular results with taxonomy. Qualitative characters considered diagnostic at the generic level were examined in specimens of *Brachidontes* under a dissecting microscope, and photographed with a Nikon D90 camera with MicroNikor 105 mm lens. Those characters include [1] the position of the umbo with respect to apical end of the valves, [2] the presence and nature of apical teeth in the hinge region, [3] the nature of the insertion area of the anterior adductor muscle, [4] the relative length of the ligament along the dorsal edge of the valves, [5] the differentiation and position of postero-lateral teeth relative to the mid-dorsal inflection point of the shell outline and the posterior end of the ligament, [6] the disposition of the anterior end of the ligament relative to the hinge plate, and [7] the presence and nature of crenulations along the border of the valves.

**Table S4.1 Phenotypic characters of *Brachidontes* spp.**

| **Species** | **Hinge plate** | **Ligament** | **Insertion area of anterior adductor muscle** | **Ventral marginal crenulation** | **Postero-lateral (dorsal) teeth** |
| --- | --- | --- | --- | --- | --- |
| *Brachidontes solisianus [[1]](#footnote-2)* | Hinge plate with a series of ~ 7-8 teeth of variable size and shape, from round an blunt to small denticles | Ligament strong, extending anteriorly along an irregular groove, between the umbo and the hinge plate | Elevated, prominent | Antero-ventral margin smooth | About 6 teeth, usually well differentiated; located at or slightly behind mid-dorsal inflection point |
| *Brachidontes darwinianus[[2]](#footnote-3)* | Two teeth followed by a short gap, and then approximately 10 small additional teeth | At least in some specimens there is a delicate posterior expansion of the hinge plate that envelops the anterior end of the ligament. The structure appears to brake down in large individuals | Flat Scar | Antero-ventral margin smooth | 7-12 well differentiated teeth; located between posterior end of the ligament and dorsal inflection point |
| *Brachidontes rodriguezii [[3]](#footnote-4)* | Row of 5–6 to up to 20 very small denticles | Extending anteriorly between the umbo and the hinge plate | Low, opaque | Ventral margin smooth | About 4-6 teeth, inconspicuous to well differentiated; located at or slightly behind mid-dorsal inflection point |

1. Based on specimens from Praia da Cima, Santa Catarina (Brazil). [↑](#footnote-ref-2)
2. Based on specimens from Punta Ballena, Maldonado (Uruguay) (one of the locations of origin for the original description) [↑](#footnote-ref-3)
3. Based on specimens from San Blas Bay, Argentina (type locality of the species). [↑](#footnote-ref-4)
